# Intricate chemosymbiosis in a widespread shallow-water thyasirid clam

**DOI:** 10.1126/sciadv.adw8163

**Published:** 2026-03-04

**Authors:** Menggong Li, Yunlong Li, Shi-Hai Mao, Zhixin Zhang, Chong Chen, Xueying Nie, Xu Liu, Hui Wang, Xiaoshou Liu, Weipeng Zhang, Qiang Lin, Guang-Chao Zhuang, Jin Sun

**Affiliations:** ^1^ Key Laboratory of Evolution & Marine Biodiversity (Ministry of Education) and Institute of Evolution & Marine Biodiversity, Ocean University of China, Qingdao 266003, China.; ^2^ Laboratory for Marine Biology and Biotechnology, Qingdao Marine Science and Technology Center, Laoshan Laboratory, Qingdao 266237, China.; ^3^ Frontiers Science Center for Deep Ocean Multispheres and Earth System, and Key Laboratory of Marine Chemistry Theory and Technology, Ministry of Education, Ocean University of China, Qingdao 266100, China.; ^4^ CAS Key Laboratory of Tropical Marine Bio-resources and Ecology, South China Sea Institute of Oceanology, Chinese Academy of Sciences, Guangzhou, China.; ^5^ X-STAR, Japan Agency for Marine-Earth Science and Technology (JAMSTEC), 2-15 Natsushima-cho, Yokosuka, Kanagawa Prefecture 237-0061, Japan.

## Abstract

Chemosynthetic symbioses between animals and bacteria are common in marine ecosystems, but the symbioses in shallow-water thyasirid clams inhabiting suboxic sediments remain understudied despite their widespread occurrence. Here, we report that the shallow-water thyasirid clam *Thyasira tokunagai*, dominant in Yellow Sea sediments, harbors sulfur-oxidizing *Sedimenticola* symbionts in pouch-like structures on the gill; the symbionts exhibit highly consistent genomic content and functionality across the region. Two phylotypes of symbionts are present, differing by a single base in the 16*S* rRNA gene while sharing key functional genes with minimal differences. Spatial metabarcoding analyses of gills showed that individuals also vary in the level of spatial heterogeneity concerning the two phylotypes. These symbionts exhibit active Calvin cycle gene expressions and close-knit host-symbiont metabolic integration. Furthermore, we estimated the capacity of dissolved inorganic carbon assimilation in the live holobiont by radiocarbon tracing (29.3 ± 8.7 nmol C·clam^−1^·day^−1^). Our findings provide the basis for understanding chemosymbiosis in thyasirid clams, highlight the potential of *T. tokunagai* as a model for studying symbiosis, and underscore the ecological significance of shallow-water chemosymbioses overall.

## Teaser

Widespread shallow-water thyasirid clams show metabolic integration with sulfur-oxidizing symbionts.

## INTRODUCTION

The close-knit association between animals and bacteria in symbiosis has been ubiquitously reported across various host groups in both terrestrial and aquatic ecosystems (*1*, *2*). Symbionts may play crucial roles in helping their hosts with nutritional acquisition and defense against pathogens, supporting development, local adaptation, speciation, and evolution (*3*, *4*). Iconic symbioses between invertebrates and chemosynthetic bacteria have been described in chemosynthesis-based marine ecosystems, best represented by deep-sea hydrothermal vents and hydrocarbon seeps. Symbiotic bacteria in these habitats oxidize reducing substances (e.g., hydrogen gas H_2_, hydrogen sulfide H_2_S, and methane CH_4_) and produce organic matter for their hosts (*5*), including siboglinid tubeworms such as *Riftia* (*6*–*8*), abyssochrysoidean gastropod and peltospirid snails (*9*–*11*), bathymodioline mussels (*12*, *13*), and vesicomyid and thyasirid clams (*14*, *15*), forming the base of diverse and flourishing communities. Chemosymbiosis also extends to shallow-water environments, such as in ciliated protists, nematodes, gutless oligochaetes, and lucinid clams associated with seagrass or mangrove sediments, as well as thyasirid bivalves inhabiting organic-rich, cold-water sediments (*16*).

Members of the bivalve family Thyasiridae inhabit diverse environments, including organic falls (such as sunken woods and whale falls), in addition to the aforementioned habitats (hydrothermal vents, cold seeps, and reducing coastal sediments) (*17*–*20*). Notably, different species of thyasirids have adapted to a wide range of different depths, from shallow water to hadal depths (*17*, *21*, *22*). The widely distributed *Thyasira gouldii* species complex is commonly found in reducing sediments of cold, shallow-water habitats around the Northern Hemisphere from eastern Canada to Europe to Asia (*17*, *18*, *23*, *24*). Thyasirid species harboring chemosymbionts have a super extensible, vermiform foot for sulfide mining, up to 30 times of the shell length (*25*). The magnetosome in *T. goudii* chemosymbionts has been proposed to assist free-living forms in locating the oxic-anoxic interface (*26*).

Thyasirid bivalves make an excellent model for investigating the evolution of symbiosis (*25*, *27*). Most host species have a single specific symbiont clade, indicating strong selection and cooperation within the holobiont (host plus its symbionts). Different hosts in the same environment may have phylogenetically distinct symbionts, even when the symbionts are functionally similar (*15*, *28*). In addition, various levels of complexity in the association with bacteria have been reported in thyasirids, from completely asymbiotic to three types of symbiotic states (i.e., type 1: episymbiotic bacteria on the surface of the gill microvilli, type 2: episymbiotic bacteria in a space delimited by the microvilli and cytoplasm, and type 3: endosymbiotic bacteria inside the cell) (*27*). So far, more than 130 species have been described in Thyasiridae, and of these 26 species are thought to be asymbiotic (*27*). Most of the chemosymbiotic thyasirids host a single bacterial symbiont, whereas two distinct symbionts were observed in the hadal species *Tartarothyasira hadalis* (*29*).

Extensive ecological surveys on the microbenthic fauna in the Yellow Sea since 1958 have consistently shown that *Thyasira tokunagai*—a constituent of the *T. gouldii* complex—is one of the dominant species, especially in the cold water mass area of the northern Yellow Sea (*30*). However, whether *T. tokunagai* harbors chemosymbionts remains unclear. Nevertheless, the closely related congener *T. gouldii* is known to harbor chemosymbionts, which it takes up via horizontal transfer from the environment, with the major chemosynthesis pathways in the symbiont having been reported (*26*, *31*). Hence, *T. tokunagai* is a promising candidate for chemosymbiosis research.

**Fig. 1.**
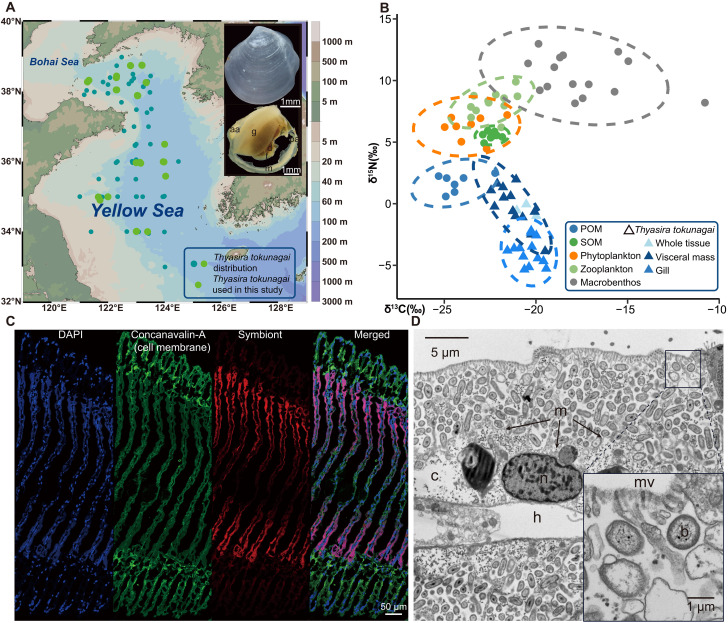
Sampling stations, stable isotope signatures, and chemosymbionts. (**A**) Shell and external anatomy of *Thyasira tokunagai*, and the distribution of the species across 162 sampling stations in the Yellow Sea based on nine research cruises (2018–2025). Specimens used in this study appear in light green. g, gill; f, foot; d, digestive diverticula; m, mantle; aa, anterior adductor; pa, posterior adductor. (**B**) Stable isotopic niche of *T. tokunagai* in the macrobenthic community of the Yellow Sea. For *T. tokunagai*, values for whole clams, gills and visceral mass tissues are represented by different colors. POM refers to particulate organic matter; SOM refers to sediment organic matter. (**C**) Fluorescence in situ hybridization showing the symbiont bacteria in the gill filaments. All cell nuclei were stained with 4′,6-diamidino-2-phenylindole (DAPI), the cell membrane was stained with Concanavalin-A, and symbionts were hybridized with the specific probe of the *T. tokunagai* symbiont *Sedimenticola* sp. (**D**) TEM micrograph of extracellular bacteria maintained in a pouch-like structure bearing microvilli. mv, microvilli; b, bacteria (the high abundance bacteria are mainly symbionts); n, nuclei; m, cell membrane; c, cell cytoplasm; h, hemocoel.

Here, we use *T. tokunagai* (Fig. 1A) as a model to study chemosymbiosis in the reducing shelf sediments of the Yellow Sea. We combine multiple research methods to investigate symbiotic features and function in *T. tokunagai*, including electron microscopy observation, barcoding and metagenomics [16*S* ribosomal RNA (rRNA) gene amplicon, spatial metabarcode, metagenome of symbionts, mitochondrial genomes of hosts, and population genetics], functional gene expression, and radioactive-based C-fixation quantification. Our findings characterize the previously overlooked chemosymbiosis in *T. tokunagai*, clarify its underlying chemosymbiotic basis, and highlight the potential of shallow thyasirids as a model for investigating marine chemosymbiotic systems.

## RESULTS

### Host-symbiont system in a cold, shallow environment

Our sampling records across nine cruises from 2018 to 2025 show that *T. tokunagai* is a common and patchily distributed benthic species in the Yellow Sea, with up to 200 specimens in a 0.1-m^2^ sampler, typically found at depths ranging from 9 to 82 m (table S1). Details of the samples used are shown in table S2. *Thyasira tokunagai* was identified from both morphology and the mitochondrial *cox1* gene barcode to confirm its placement in the *T. gouldii* species complex (Fig. 1A and fig. S1). Pairwise comparisons of the *cox1* gene of specimens collected from nine sampling locations in the Yellow Sea revealed an average of 99.84% similarity (fig. S2, A and B), and the haplotype network (fig. S2C) also showed that nearly all haplotypes lacked a clear geographical affinity. Furthermore, our STRUCTURE analysis also showed a lack of population differentiation using the alignment of 13 protein-coding genes of 30 mitochondrial genomes based on the delta *K* (*K* = 2; fig. S2D). These results indicate a panmixia condition for all nine populations of *T. tokunagai* sampled in the Yellow Sea.

Compared to other benthic fauna and environmental samples from the Yellow Sea (*32*), the δ^13^C value of *T. tokunagai* (−20.52 ± 0.43‰, *n* = 3, whole individual) was higher than that of the particulate organic matter (POM), sediment organic matter (SOM), phytoplankton, and zooplankton but lower than that of other benthic fauna (POM: −24.31 ± 1.01‰, SOM: −22.42 ± 0.36‰, phytoplankton: −23.61 ± 1.06‰, zooplankton: −22.38 ± 0.88‰, and macrobenthos: −17.54 ± 2.41‰; Fig. 1B and table S3). Unlike other species relying on chemosymbionts exclusively and having a lower δ^13^C value in symbiotic organs, a higher δ^13^C level in the symbiont-hosting gills (−20.38 ± 0.61‰, *n* = 13) compared to the visceral mass (−21.22 ± 0.89‰, *n* = 13) was observed. These results indicate that *T. tokunagai* does not entirely rely on filter feeding but may adopt a mixotrophic strategy, obtaining organic matter from sediments via deposit feeding and symbionts via chemosymbiosis. *Thysaira tokunagai* exhibited the lowest δ^15^N value (−0.23 ± 0.22‰, *n* = 3) (POM: 1.71 ± 0.71‰, SOM: 5.52 ± 0.31‰, phytoplankton: 6.20 ± 0.91‰, zooplankton: −8.16 ± 0.92‰, and macrobenthos: −10.36 ± 1.56‰; Fig. 1B and table S3). Notably, there were lower δ^15^N levels in the symbiont-hosting gills (−3.91 ± 1.08‰, *n* = 13) than the visceral mass (−0.21 ± 1.68‰, *n* = 13), indicating that symbiont-containing gills act as the initial interface for symbiont-mediated nitrogen acquisition, with nitrogen subsequently transferred to other tissues such as the visceral mass.

A bacterial species belonging to the genus *Sedimenticola* dominated the microbial community in the gills of *T. tokunagai* (fig. S3A), determined through full-length 16*S* rRNA gene amplicon sequencing, which accounted for an average of 92.34% of the bacterial community (*n* = 21, from seven sampling stations during three cruises from 2020 to 2021). The presence of *Sedimenticola* was further validated in another 99 individuals from 23 stations sampled between 2020 and 2024 by targeting the 16*S* V3-V4 region using amplicon sequencing; the symbiont represented, on average, 88% of the whole gill-bacterial communities (fig. S3B). The bacterial community in most specimens (*n* = 95) consisted of at least 60% *Sedimenticola*, with only four exceptions. Phylogenetic reconstruction using the 16*S* rRNA gene confirmed that its closest relative was the chemosymbiont of *T. gouldii* in the same host species complex (fig. S4). Notably, *Sedimenticola* was not recovered in analyses of the surrounding sediment (fig. S3C). To investigate the distribution of symbionts within the gill tissue, we conducted fluorescent in situ hybridization (FISH) imaging and showed that (i) the symbionts were concentrated in the bacteriocytes located at the middle part of the gill filament and absent from the ciliated filament tip and (ii) symbionts seemed to be enveloped by a layer of membrane (indicated by the green signal) (Fig. 1C and fig. S5). Nonetheless, transmission electron microscopy (TEM) observation (Fig. 1D) of the gill tissue showed that symbionts were actually localized in extracellular pouch-like structures among the microvilli but not completely enclosed in vesicles, implying an exocellular symbiotic mode where bacteria are maintained outside of the host cytoplasm but in a specialized pouch-like organ (*27*).

### Two symbiont phylotypes

**Fig. 2.**
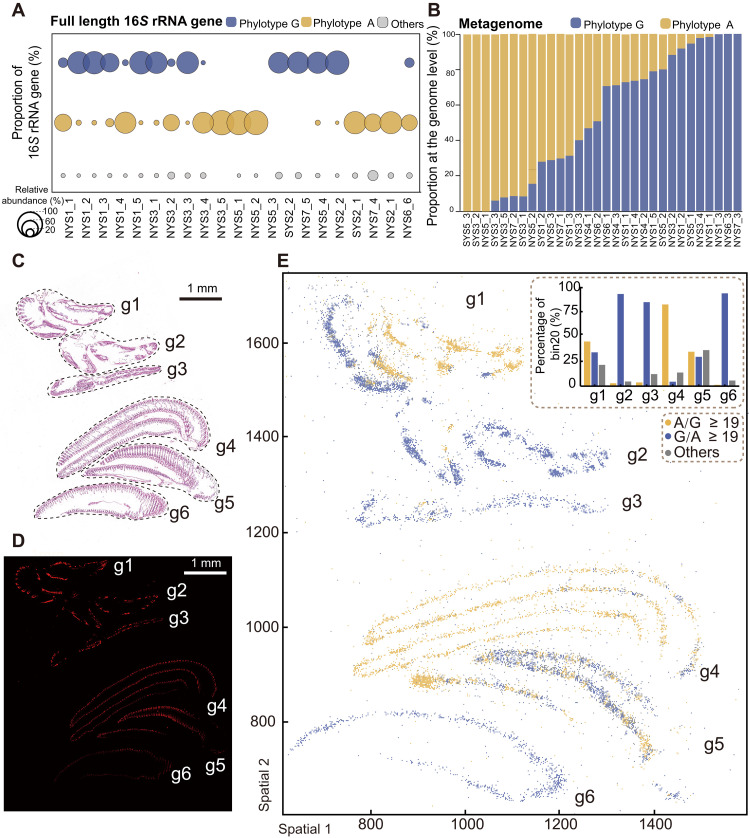
Identification and spatial distribution of the two *Sedimenticola* symbiont phylotypes in *Thyasira tokunagai* gill tissue. (**A**) Two dominant phylotypes belonging to the genus *Sedimenticola* were identified in gill tissue bacterial communities, which we call *Sedimenticola* sp. (ex *T. tokunagai*) “phylotype A” and “phylotype G” based on the full-length 16*S* rRNA gene amplicon sequencing, differing by a single base at the 590th position; the first 13 samples were used for metagenomic sequencing, while the last 8 were used for metatranscriptomic sequencing. NYS represents the sampling stations from the north Yellow Sea, whereas SYS represents the sampling stations from the south Yellow Sea. (**B**) Strain decomposition analysis revealed the presence of two symbiont phylotypes, corresponding to the two dominant phylotypes in genome-level identified in (A). (**C**) An H&E-stained gill tissue section (scale bar, 1 mm) with consecutive sections used for FISH imaging (D) and spatial phylotype distribution analysis (E). (**D**) FISH imaging with the symbiont-specific probe showing the distribution of symbionts in a gill tissue section (scale bar, 1 mm). (**E**) At the cellular level, the spatial distribution of symbiont phylotypes is such that each spot represents a 10 μm bin, named “bin 20”, approximating the size of a single cell, and the A/G ratio indicates the relative abundance of phylotype A compared to phylotype G at each location (e.g., A/G > 19 indicates that phylotype A is ~19 times more abundant than phylotype G within that bin).

Two phylotypes of the same *Sedimenticola* species made up the bacterial population in *T. tokunagai* (Fig. 2A and fig. S3D). There was only a single base pair difference (G versus A) between these two phylotypes at the 590th position of the 16*S* rDNA, verified by Sanger sequencing (note S1)—each phylotype accounting for 46.29 and 45.88% of the overall symbiont population (mean percentage), respectively (Fig. 2A). Host individuals differ greatly in the proportion of the two phylotypes in 16*S* rRNA gene and metagenomic datasets, showing a whole range including some with only one or the other phylotype (Fig. 2B and fig. S6). Spot analyses (10 μm in diameter) of gills showed that more than 80.59% of 10,468 spots exhibited just one phylotype, indicating that there is a bias to hosting just one phylotype within each symbiotic pouch. Hematoxylin and eosin (H&E) imaging and FISH analysis confirmed the symbiont distribution pattern on the gill filament (Fig. 2, C and D). Spatial analyses of gills showed that individuals also vary in the level of spatial heterogeneity concerning the phylotypes (each two gills from six individuals; Fig. 2E and fig. S7)—especially in the individual g5 showing a well-mixed pattern of the two phylotypes.

**Fig. 3.**
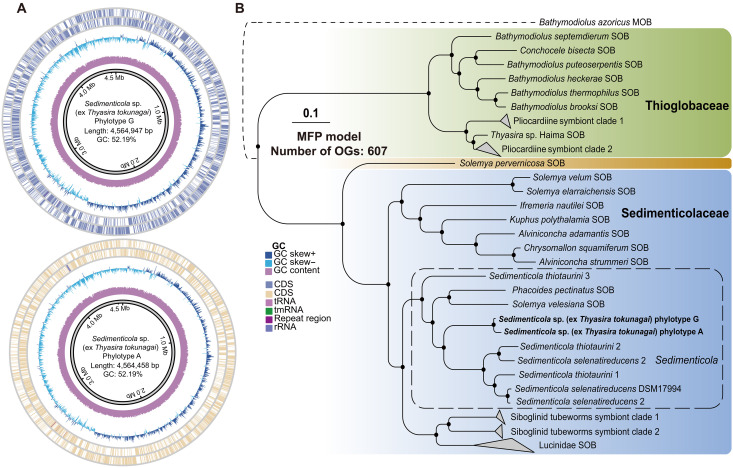
Phylogenetic relationships of the two *Sedimenticola* symbiont phylotypes. (**A**) General features of the circular symbiont genomes in the two phylotypes, showing a similar genome size (a total of 4.5 Mb) and a GC content of 52.19%. (**B**) Phylogenomic analysis, rooted with the methanotrophic (MOB) symbiont of the hot vent bathymodioline mussel *Bathymodiolus azoricus*, demonstrates that the two phylotypes fall within the family Sedimenticolaceae (SOB) and are closely related to cultured *Sedimenticola* species (solid black dot at the node indicates 100 bootstrap supports; scale bar, 0.1 substitutions per nucleotide site). The phylogenetic tree was inferred using IQ-TREE with the maximum likelihood (ML) method under the best-fit model automatically selected by ModelFinder and 1000 ultrafast bootstrap (-m MFP, -B 1000) based on a concatenated matrix of 607 genes. tRNA, transfer RNA; tmRNA, transfer-messenger RNA; CDS. coding sequence.

Combining long-read and short-read sequencing, a total of 30 high-quality circular genomes [i.e., metagenome-assembled genome (MAGs)] were assembled, with completeness >99.23%, contamination rate < 0.34%, and size of 4.5 Mb (Fig. 3A and table S4). The average nucleotide identity (ANI) of these 30 MAGs ranges from 98.90 to 99.94% (fig. S8 and table S5), well above the proposed threshold of interspecies variation of prokaryotes (95%) and supports them as belonging to the same species (*33*). A representative genome of each phylotype was further deduced using StrainPanDA (Fig. 3A). Genome capacity analysis revealed no key functional gene differences related to chemoautotrophy between the two phylotypes. Phylotype A-specific genes include 3-hydroxyacyl–coenzyme A dehydrogenase, nicotinamide adenine dinucleotide (NAD)–binding domain-containing proteins, and multiple hypothetical proteins. In contrast, phylotype G–specific genes encompass transposase family proteins, poly-beta-hydroxybutyrate polymerase, redox-regulated molecular chaperones, toxin-antitoxin system proteins, formylglycine-generating sulfatase enzymes, and hypothetical proteins (table S6). To boost the credibility of our findings, we conducted a correlation analysis. This analysis revealed a positive correlation between the percentage of phylotype G from StrainPanDA and the percentage of metagenomic reads of the base G at the 590th position in the 16*S* rRNA gene (*R*^2^ = 0.97; fig. S9). Placement in the genus *Sedimenticola* was also shown by phylogenetic reconstruction at the genomic level (Fig. 3B and table S7). Overall, it seems that the two phylotypes are equivalent in function, and the host individuals do not actively select for one or the other phylotype, instead the two are being used interchangeably.

### Metabolic capacities of host and symbiont

**Fig. 4.**
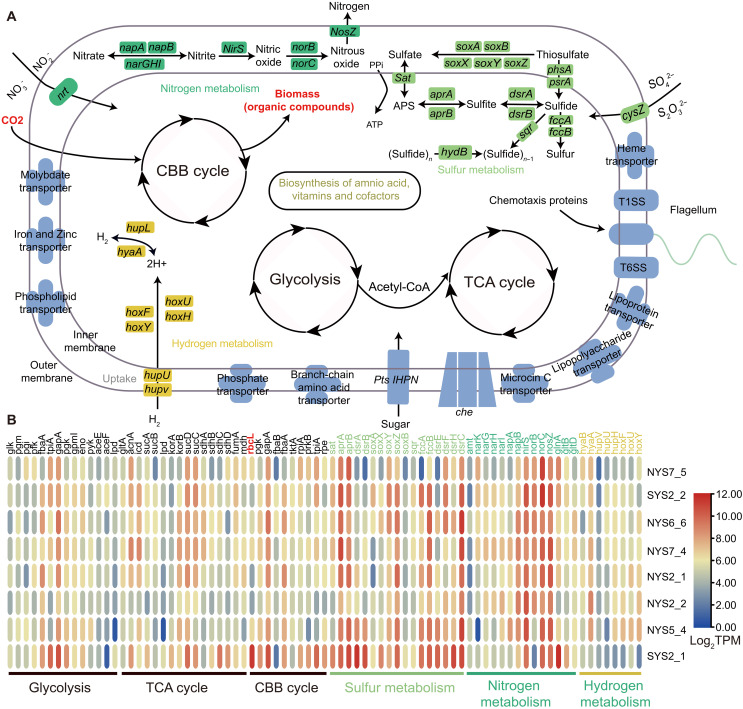
Chemosynthetic capacities and transcriptome profiles of symbiont phylotypes. (**A**) Reconstructed chemosynthesis pathways from both symbiont genomes indicate a conserved metabolic potential, consistent with a free-living lifestyle. Both phylotypes encode a complete set of enzymes for carbon fixation and utilization and have the potential to utilize sulfides and hydrogen as energy sources. (**B**) Transcriptome profiles of highly conserved chemosynthetic pathways related to carbon, sulfur, nitrogen, and hydrogen metabolism. Transcribed functional genes were annotated against the KEGG database, and expression levels are shown as log_2_-normalized transcripts per kilobase million (TPM). CoA, coenzyme A.

The metabolic potential of the *Sedimenticola* symbiont of *T. tokunagai* was highly conserved (Fig. 4A), highly similar to the published result in the closely related *T. gouldii* (*34*). They encode the full set of enzymes in carbon fixation and utilization, including the Calvin-Benson-Bassham cycle (CBB cycle or the reductive pentose phosphate cycle), glycolysis/gluconeogenesis, tricarboxylic acid cycle (TCA), and oxidative phosphorylation, enabling both phylotypes to assimilate dissolved inorganic carbon. The type II ribulose-bisphosphate carboxylase (*rbcL*, K01601) in the Calvin cycle is responsible for the assimilation of inorganic carbon. The reductive TCA cycle cannot function completely in the *T. tokunagai* symbiont due to the absence of type II adenosine 5′-triphosphate (ATP) citrate lyase, unlike the symbiont of the giant tubeworm *Riftia pachyptila* (*35*). The complete dissimilatory nitrate reduction pathway allows respiration under an anaerobic or hypoxic environment, while, due to the lack of the nitrite reductase (reduced form of nicotinamide adenine dinucleotide), large subunit (K00362) hinders it from producing ammonia. A complete dissimilatory sulfate reduction pathway and a partial sulfur oxidation (SOX) system were also found, mainly containing *soxA*, *B*, *X*, *Y*, and, *Z*, but *soxCD* was lacking. An incomplete assimilatory sulfate reduction pathway was detected, which contained *sat* and *cysC*.

In five sediment cores, oxygen penetration depth varied from 3.2 to 4.7 mm, which was shallower than the layers where *T. tokunagai* were found (1 to 7 cm; fig. S10, A and B). Although oxygenated seawater is brought into the mantle cavity, we noted the active expression of genes involved in nitrate reduction in *T. tokunagai* symbionts, including *narG*, *H*, *I*, *norB*, *C*, *napA*, *B*, *nirS*, and *nosZ* (Fig. 4B). We also found the genes and enzymes related to hydrogen oxidation, such as *hoxF*, *U*, *Y*, *H*, *hybC*, and *hyaB*. The symbiont encodes ABC transporters and PTS pathways, indicating the capacity for heterotrophy. Bacterial chemotaxis and flagellar assembly pathways were found. In addition, both phylotypes have a relatively complete capacity for the biosynthesis of amino acids (*20*), vitamins, and cofactors (*10*) (table S8), suggesting that they have the capacity for a free-living life style. While not expressed at a high level across all individuals, genes in these pathways (CBB cycle, sulfur oxidation, and nitrogen metabolism) appeared to be active in at least some host individuals at the time of sampling (Fig. 4B).

For core nutrient cycling, the symbiont exhibited high completeness in carbon fixation pathways (e.g., CBB cycle), sulfur metabolism (e.g., dissimilatory sulfate reduction), and nitrogen metabolism (e.g., denitrification), whereas these pathways were functionally incomplete in the host genome (Fig. 4A and fig. S11). This discrepancy indicates the symbiont not only undertakes inorganic carbon fixation and sulfur cycling to supply the host with organic carbon and reduced sulfur compounds but may also use nitrogenous waste products generated by the host. In amino acid and vitamin biosynthesis, the symbiont harbored fully complete pathways for synthesizing most amino acids and vitamins (e.g., thiamin, riboflavin, and cobalamin), for which the host genome did not contain complete pathways, indicating that the symbiont may provide essential organic nutrients to the host. Metabolic exchange is further supported by the presence of transporters (e.g., vitamin B12 and phosphate transporters) in both partners and intact bacterial secretion systems in the symbiont—implying that the symbiont can actively secrete metabolites, which are then taken up by the host via transporters. Collectively, these patterns demonstrate a reciprocal metabolic relationship which the symbiont relies on the host’s micro-niche (i.e., burrowing deep in sediment to access sulfides) and uses host-derived metabolic waste (e.g., nitrogenous compounds) while supplying the host with carbon, nutrients, and cofactors; the host, in turn, supports the symbiont and acquires essential metabolites.

### Carbon fixation rate measurement

**Fig. 5.**
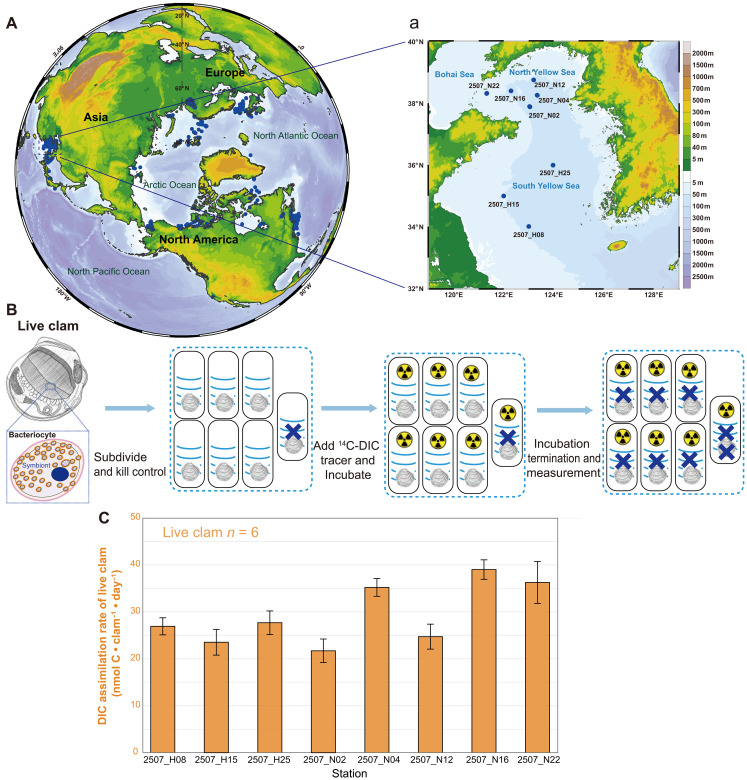
Global distribution of *Thyasira gouldii* complex and the measurement of DIC assimilation rate of *T. tokunagai.* (**A**) Global distribution of *T. gouldii* complex and (a) the distribution of *T. tokunagai* (live clam) used for DIC assimilation determination in the Yellow Sea. (**B**) Schematic of the ^14^C-labeled DIC assimilation assay method. To assess the DIC assimilation rate, ^14^C-labeled DIC tracer was added to live clam individuals, and the individuals were incubated at simulated in situ temperatures. The “X” symbol atop live clams represents the individuals being killed with formaldehyde. (**C**) DIC assimilation rates of live clams (*n* = 6). Live clam assimilation rates are expressed as nanomoles of carbon assimilated per clam per day.

The *T. gouldii* complex, which *T. tokunagai* is a part of, is widely distributed across the entire northern hemisphere (Fig. 5A). A key point is how much carbon could be transferred from inorganic to organic forms. We used ^14^C-labeled dissolved inorganic carbon (DIC) tracers to estimate the carbon assimilation rate constant (*k*) of whole, live thyasirid individuals (eight stations, replicates = 6) under ambient (in situ) temperatures and incubated using in situ sterile seawater collected from the same locality taken at the same time when the clams were sampled. The experimental design is shown in Fig. 5B and fig. S12A, with further details and in situ environmental parameters shown in table S9 and fig. S13. The microbiota in the seawater (eight stations, replicates = 3) assimilated relatively little carbon, ranging from 2.10 × 10^−5^ to 5.13 × 10^−5^ (*k*, day^−1^). The DIC assimilation rate of seawater was 8.43 ± 2.69 nmol C·100 ml^−1^·day^−1^ (calculated under the in situ DIC levels; fig. S12B). Comparatively, higher rate constants were observed in the live *T. tokunagai* holobiont, with the mean level of 7.4 × 10^−4^ (from 3.70 × 10^−4^ to 1.43 × 10^−3^), up to 21 times as high as the seawater values. When calculated with the DIC concentrations of stations, the DIC assimilation rate of a single thyasirid holobiont ranged from 13.0 to 55.7 nmol C·clam^−1^·day^−1^, with the mean and SD level of 29.3 ± 8.7 (calculated under the in situ DIC levels; Fig. 5C). The carbon fixation capacity of a single thyasirid is thus approximately comparable to that of 370 ± 142 ml of the surrounding seawater (from 160 to 871 ml, 48 thyasirids and 24 seawater samples from eight stations). Meanwhile, the carbon assimilation rates in whole holobionts were lower than the homogenized gill tissue incubations (fig. S14). Specifically, the maximum carbon assimilation rate in homogenized gill tissue solutions was 195.6 nmol C·day^−1^ per clam (specimens from station N04, 202407 cruise, incubated at 20°C), ~3.5-fold higher than that of the whole holobionts (55.7 nmol·day^−1^ per clam, specimens from station N22, 202507 cruise, incubated at 6°C).

Temperature is one of the key factors affecting enzyme activity in animals. At four stations (fig. S15A), we added ^14^C-labeled DIC tracers to homogenized gill tissues from four individuals combined to account for individual differences, to investigate the effect of temperature on carbon assimilation (table S9; experimental design in fig. S15B). The homogenized gill samples from each station were incubated at four temperatures (i.e., 5°, 12°, 20°, and 28°C), covering the range of yearly temperatures measured in the natural habitat (fig. S15C). Results showed that the carbon assimilation rate constant (*k*, day^−1^) was lowest at 5°C across all stations (average: 2.15 × 10^−3^ ± 1.37 × 10^−3^). As the temperature increased, *k* exhibited higher levels, with the highest and comparable values at 12° and 20°C (5.09 × 10^−3^ ± 0.45 × 10^−3^ and 4.79 × 10^−3^ ± 0.98 × 10^−3^, respectively). A declining trend was observed with further increase in temperature up to 28°C (*k* = 3.51 × 10^−3^ ± 1.20 × 10^−3^), where *k* was equal to 69% of the highest value. Nonetheless, symbionts in *T. tokunagai* appear to convert more DIC to organic matter at elevated temperatures. A cubic regression model presented a better representation of measured values than a quadratic model (fig. S15C), predicting a peak assimilation rate constant at 14.78°C, which decreases under both warmer and cooler conditions.

## DISCUSSION

### Deciphering thyasirid trophic strategies using isotopic signatures

Stable carbon and nitrogen isotopic data provide key insights into the trophic strategy of *T. tokunagai*. For carbon isotopes, obligately chemosymbiotic bivalves typically exhibit significantly lower δ^13^C values in gill tissues (usually <−26‰) than in nonsymbiotic tissues, a pattern directly linked to their high reliance on symbiont-derived carbon (*36*). In contrast, the δ^13^C values of *T. tokunagai* are closer to the isotopic range of phytoplankton-derived organic carbon, and its gill tissues showed slightly higher δ^13^C values than the visceral mass on average. This suggests *T. tokunagai* adopts a mixotrophic strategy with low dependence on chemotrophy and is also consistent with findings on the closely related *T*. cf. *gouldi* (*37*). It was also observed that both symbiotic and asymbiotic thyasirids had gill δ^13^C values close to −20‰; notably, symbiotic thyasirids did not exhibit the very low δ^13^C signature characteristic of other obligately symbiotic bivalves, potentially influenced by exogenous carbon [e.g., suspended particulate organic matter (SPOM)] (*37*). *Thyasira tokunagai* symbionts have type II RuBisCO, and δ^13^C values associated with type II RuBisCO are generally higher than those with type I (*38*). This also explains the relatively high carbon isotope signal of *T. tokunagai* (~−22.87 ± 1.16‰) and supports the inference that symbiont-derived carbon makes a limited contribution, while exogenous carbon (e.g., phytoplankton) accounts for a large proportion of its carbon intake. In addition, Cardini *et al.* (*39*) studied lucinid bivalves in seagrass beds and found seasonal fluctuations in δ^13^C differences between symbiont-hosting and symbiont-free tissues. These differences may arise from variations in the magnitude of chemosynthesis-derived nutrient inputs or habitat-specific environmental regulations.

For nitrogen isotopes, Zanzerl *et al.* (*37*) showed that symbiotic thyasirids had significantly lower δ^15^N values in gill tissues (as low as −5.88‰), resulting from symbionts’ preferential use of reduced nitrogen sources with light δ^15^N in sediments. In contrast, asymbiotic thyasirids exhibited higher and less variable δ^15^N values due to their reliance on SPOM or free-living sulfur-oxidizing bacteria. Light nitrogen sources fixed by symbionts and exogenous nitrogen together constitute the nitrogen input to nongill tissues. This is in line with the mechanism proposed by Dufour *et al.* (*25*), wherein thyasirid symbionts regulate chemosynthesis based on sulfide availability. Sulfide modulates symbiont activity to alter nitrogen metabolism efficiency, indirectly influencing nitrogen isotope fractionation. This ultimately results in mixed nitrogen input combining symbiont-derived and exogenous nitrogen, a characteristic consistent with the mixotrophic strategies observed in both Zanzerl *et al.* (*37*) and our study.

### Chemosynthetic capacity of thyasirids

Chemosymbiosis is widely distributed across many animal groups, but most studies have been focused on annelids and mollusks from “extreme” deep-sea ecosystems such as vents and seeps (*5*, *40*), which are difficult to maintain in the laboratory for experimentation. In contrast, many thyasirid bivalves are widely distributed in suboxic mud outside vent and seep systems, including those in the shallow-water; but remain understudied and have poorly known ecological roles. Our study presents a detailed investigation of the symbiotic association in *T. tokunagai*, revealing the dominance of a sulfur-oxidizing bacteria (SOB) symbiont belonging to *Sedimenticola* in their gills with close affinity to other previously reported chemosymbionts of other animals (fig. S3, A and B).

Consistent with previous metagenomic work on the closely related congener *T. gouldii* (*34*), the *T. tokunagai* symbiont genomes also exhibited *soxA*, *B*, *X*, *Y*, and *Z*, but not *soxCD*. The absence of *soxCD* would lead to the partial oxidation of thiosulfate and the accumulation of zero-valent sulfide, forming sulfur globules in the symbionts, which are common in SOB such as *Erythrobacter flavus* (*41*) and *Chlorobaculum limnaeum* (*42*). Sulfur globule-like structures were observed in the symbiont of both *Thyasira flexuosa* and *T. gouldii* under TEM (*28*, *43*). On the other hand, genomes of our *T. tokunagai* symbiont contained a complete dissimilatory sulfate reduction (*dsr*) pathway, suggesting the genetic basis to fully oxidize sulfide to sulfate (*44*). During that process, ATP is generated by sulfate adenylyltransferase (*sat*) and then used in the Calvin cycle to fix CO_2_, which is a central principle of chemosymbiosis. In addition, *T. tokunagai* symbionts appear to be capable of fixing inorganic carbon via oxidizing reducing substances, with the SOX system, reverse dissimilatory sulfate reduction pathway, and the HOX system (*45*, *46*). Similar to previous research, nitrate might be the electron receptor for symbiont respiration due to the oxygen-limited sediments where these thyasirids live (*15*). The DIC assimilation has been quantified, providing a robust support for chemosymbiosis in *T. tokunagai*.

### Strong selection by the host on the symbiont and apparent horizontal transmission

Although our sampling stations of *T. tokunagai* covered a wide area in the Yellow Sea, with the longest distance from the southernmost point to the northernmost point exceeding 500 km, there was an undifferentiated, panmictic population of *T. tokunagai* across all stations as evidenced by mitochondrial population genomics. The symbiont population in the gill of *T. tokunagai* is greatly dominated by a single symbiont species (genus *Sedimenticola*), pointing to strong selection of symbionts by the host (*47*). The ratio of the two phylotypes within each host likely results from the local bias within each symbiont-hosting pouch (spatial metabarcoding result in Fig. 2E and figs. S6 and S7). Overall, we observed a lack of bias in phylotype uptake by the host, with a comparable percentage of the two phylotypes across the whole *T. tokunagai* populations and, in some individuals, the presence of both phylotypes, often spatially segregated, in a single gill. The two phylotypes differ by only one base pair in the 16*S* rRNA gene and appear to largely lack functional differentiation, supported by the highly similar gene sets between the two deduced phylotypes’ genomes. This is different from the symbiont phylotype diversity in deep-sea bathymodioline mussels, affecting host fitness (*48*). Our results suggest all individuals of *T. tokunagai* share symbionts of an overall consistent function and ecology, enabling extrapolation of measured carbon fixation rates to the whole Yellow Sea population. Although the two symbiont phylotypes exhibit high genomic similarity, they harbor subtle functional differences that could contribute to variation in carbon fixation rates among host individuals.

Previous studies reported the super extensile, vermiform foot in thyasirid bivalves is used to mine sulfide and potentially form a means to acquire magnetosome-containing symbionts (*25*, *26*), implying that the symbionts might be obtained from a specific niche of sediments. The metabolic potential of the symbiont genomes, with the capacity for heterotrophy and free-living existence, is suggestive of horizontal transmission. Meanwhile, the spatially resolved pattern of two phylotypes within individual gills might be a consequence of “who settles first wins,” which fits the horizontal transmission mode. Therefore, we consider *T. tokunagai* most likely acquires the *Sedimenticola* symbionts from the environment via horizontal transmission, acquired under the control of a highly selective mechanism by the host clam.

### Potential factors determining carbon fixation rates in thyasirid holobionts

Chemosymbiotic animals are recognized as “bacterial reservoirs.” Deep-sea hydrothermal vent mussels such as *Bathymodiolus puteoserpentis* often have far larger body sizes than *T. tokunagai*, while hosting symbiotic bacteria that occupy ~50% of the bacteriocyte volume—showing that chemosymbiotic bivalves, regardless of body size, concentrate bacteria at densities far higher than their surrounding environments (*49*). Previous research has leveraged sequencing to uncover the potential of chemosynthetic carbon fixation through the presence or expression level of functional chemosynthetic genes. The capacity and the assimilation rates of inorganic carbon by chemosymbiotic species have been rarely investigated by experiments with precisely controlled variables, with only a few studies using controlled incubations. Petersen *et al.* (*50*) quantified the ^14^C activity in the gills of the deep-sea mussel *B. puteoserpentis* to validate the hypothesis that hydrogen serves as energy for hydrothermal vent symbioses. Similarly, the carbon fixation rates of the awning clam *Solemya velum* symbionts were found to be affected by pH, sulfide, and oxygen (*51*). Cardini *et al.* (*39*) followed the fate of carbon and nitrogen in shallow-water lucinid clams and estimated a carbon fixation rate of 300 to 2600 nmol C·g gill tissue^−1^·hour^−1^. Compared to this data from shallow-water lucinid clams (*39*), *T. tokunagai* exhibits lower DIC assimilation (4.2 to 116.1 nmol C·g clam^−1^·hour^−1^), with its maximum rate being 4.5% of lucinids. However, normalizing by gill tissue weight may reduce these intertaxa differences (see fig. S16). Potential factors contributing to this observed difference in carbon assimilation rates may include variations in incubation temperature, symbiont abundance, symbiont-derived differences in dissolved inorganic carbon utilization efficiency, and carbon consumption by the host.

Previous studies used excised gills of homogenized symbionts, rather than the organic carbon consumption of the entire host. Here, we present an attempt to estimate the rate of DIC assimilation targeting the entire holobiont (host plus its chemosymbionts) via radiocarbon (^14^C) uptake under in situ temperatures and bottom seawater (filtration sterilization). Our results revealed the DIC assimilation capacity in the thyasirid, where the holobiont as a whole had lower rates than the homogenized gill with symbionts. We acknowledge that our current experimental design could not fully replicate in situ conditions—where the clam is buried in its burrow and modulating the chemical microenvironments for symbionts—due to the restrictions on radioactive substance transport during cruises and the inability to restore the vertical distribution of clams in sediments. This may lead to a potential underestimation of the carbon fixation rates of the *T. tokunagai* holobiont. Meanwhile, differences between trials (i.e., live clams) could have been related to the proportion of time these clams spent with the valves shut. Host behavior and metabolism can also explain some of the differences observed between live clam and gill homogenate incubation results at similar temperatures (*31*).

Microbial populations in the suboxic sediments surrounding those thyasirids are potential contributors to dark carbon fixation (DCF) in the Yellow Sea, having a greater importance than bottom seawater microbes (*52*). The estimated contribution of thyasirid holobionts to carbon fixation is low in comparison to the contribution of sedimentary microbial communities. The stark difference between sediment and seawater DCF rates may be due to sediments providing abundant reductive substrates (e.g., sulfide) and micro-niches for chemoautotrophs, whereas seawater lacks comparable concentrations of these electron donors to support high chemosynthetic activity. Therefore, sulfide availability may be a key environmental driver linking both *T. tokunagai*’s DIC assimilation and bulk sedimentary DCF. Data demonstrating a strong positive correlation between sediment sulfide content and DCF rates (*52*) further indicate that *T. tokunagai*’s symbiont-mediated DIC assimilation is also dependent on sulfide. In addition, when comparing DIC assimilation across taxa, key factors to consider include the relative size of symbiont-hosting gills and environmental conditions (e.g., incubation temperature).

Collectively, we used multi-omics and radiotracer techniques to elucidate the interplay between host and symbiont in a shallow-water thyasirid holobiont, providing the first estimate of thyasirid carbon fixation rate (a rough estimation of the carbon fixation flux is included in the Supplementary Materials). Our findings showed the potential of *T. tokunagai* as a previously unidentified model in studying chemosymbiosis that allows for experimental manipulation. Further ecological investigation into the distribution and density of these chemosymbiotic organisms is sought, along with in-depth studies of the factors influencing their carbon fixation rate.

## MATERIALS AND METHODS

### Sampling description

*Thyasira tokunagai* (Fig. 1A) individuals were collected from 107 of 162 sampled stations in the Yellow Sea between 9 and 80 m in depth during nine cruises on-board the R/V *Lanhai 101* from 2018 to 2025, with the station details, environmental parameters, and specimens used in this study shown in tables S1 and S2. A 0.1-m^2^ box corer was used to collect the surface sediments. The *T. tokunagai* individuals were manually picked out from the sediments via a 0.5-mm sieve once they were on board, and some were immediately fixed, while the other live clams were maintained in low-temperature recirculating water tanks containing in situ sediment at ~10°C. For details on the sample preservation, please see the Supplementary Materials.

### Stable isotope analysis

Stable isotope analysis of carbon (C) and nitrogen (N) was conducted as previously described (*53*). The whole body, gill, and visceral mass tissues of *T. tokunagai* were freeze-dried for several hours at −60 °C until completely dry, and ~0.1 mg of the powdered sample was analyzed for stable isotopes using an isotope ratio mass spectrometer (Sercon Instruments, Crewe, UK) at the Third Institute of Oceanography, China. The carbon isotope abundance ratio was calculated using the international standard Vienna Peedee Belemnite to determine the δ^13^C value, with an analytical precision of ±0.2‰. Similarly, the nitrogen isotope abundance ratio was based on atmospheric nitrogen to calculate the δ^15^N value, with an analytical precision of ±0.25‰. All stable isotopic data in the Yellow Sea can be found in table S3 (*32*).

### Transmission electron microscopy

Gill tissues were fixed overnight using a solution containing 2.5% glutaraldehyde and 2% paraformaldehyde (PFA) in phosphate-buffered saline (PBS). The tissue was washed in 0.1 M PBS three times for 15 min each and then fixed with 1% osmium tetroxide (OsO_4_) for 1 hour, followed by additional wash using PBS. It was dehydrated through a methanol series (50, 70, 90, and 100%, three times for 15 min each) and embedded in Epon 812 resin. Ultrathin sections (70 nm) were sliced using a Reichert ULTRACUT slicer (Austria) and stained with uranyl acetate and lead citrate double staining method (*54*) for 15 min each. Images were captured by a JEM-1200EX (JEOL, Japan) TEM at an accelerating voltage of 80 kV.

### FISH and histology

For FISH experiments, the symbiont-specific probe labeled with CY5 (5′-TCCTCTATCACACTCTAGCTCAGCAGTATC-3′), the sense probe labeled with CY3 (5′-GATACTGCTGAGCTAGAGTGTGATAGAGGA-3′), and the bacterial universal probe EUB338 labeled with CY5 were designed on the basis of the corresponding representative 16*S* rRNA gene (*55*). The respective 16*S* rRNA gene of symbionts was selected for probe design, a 30-base oligonucleotide sequence was selected from the variable region of the 16*S* rRNA gene. To ensure that the probe was unique and specific, the selected probe sequence was searched against the custom database [i.e., the amplicon sequence variant (ASV) files generated from 16*S* rRNA gene amplicon]. Meanwhile, the uniqueness of the probe sequences for the symbiont was further confirmed by searching against the National Center for Biotechnology Information (NCBI) nucleotide (NT) database. Gill tissues (fixed in PFA and preserved in pure methanol) were dehydrated in 100% methanol for 30 min each, embedded in paraffin, and then sectioned into 7-μm-thick slices using a semiautomatic microtome (Leica, Germany). After removing paraffin with xylene and ethanol, the sections were rehydrated in a decreasing ethanol series (100, 95, 80, and 70%) for 15 min each, followed by hybridization at 46°C with a hybridization buffer [working concentration: probe (5 μg/ml) in 0.9 M NaCl, 0.02 M tris-HCl, 0.01% SDS, and 30% formamide] for 1 hour. Following hybridization, the slides were washed in a washing buffer (0.1 M NaCl, 0.02 M tris-HCl, 0.01% SDS, and 5 mM EDTA) at 48°C for 5 min each, and subsequently, the cell nucleus and cell membrane were stained with 4′,6-diamidino-2-phenylindole (Solarbio) and Alexa Fluor 488 conjugate Concanavalin-A (Invitrogen, CA, USA) for 10 min at room temperature, respectively. After washing using PBST (Tween 20:PBS = 1:1000), the slides were mounted with ProLong Diamond Antifade Mountant (Invitrogen). Images were captured using a ZEISS LSM 900 or Andor Dragonfly 302 confocal laser scanning microscope. For hematoxylin and eosin (H&E) staining, dewaxed tissue sections were stained according to standard protocols. Subsequently, sections were dehydrated and mounted with neutral balsam. Images were captured by a pathological section scanner (Leica SDPTOP HS6).

### 16*S* rRNA gene amplicon sequencing

Genomic DNA was extracted from the gill tissues using the DNeasy Blood & Tissue Kit (QIAGEN, Hilden, Germany), following the manufacturer’s protocol. DNA was also extracted from ~0.5 g of ambient surface sediments (wet weight) by using the PowerSoil DNA Isolation Kit (QIAGEN, Hilden, Germany). NanoDrop Lite (Thermo Fisher Scientific, USA) and 1% agarose gel electrophoresis were used to check the DNA quantity and quality, respectively. Full-length 16*S* rRNA gene of bacteria from 6 sediment and 21 *T. tokunagai* gill samples were also amplified by the primers 27F and 1492R (*56*). High fidelity (HiFi) reads were generated from the PacBio RS II platform by Novogene (Tianjin, China) with CCS mode in gill samples and ambient sediment samples, respectively. In addition, the V3-V4 region of the same gene was amplified from 99 gill samples using primers 341F and 805R (*57*), and short reads were generated from the Illumina NovaSeq 6000 platform with paired-end mode and a read length of 150 base pairs (bp) by Novogene (Tianjin, China). QIIME version 2023.9.1 (*58*) was used to process data with the standard pipeline, containing quality control, ASV table construction, and taxonomic classification. ChiPlot (www.chiplot.online) was used to visualize the bacterial community composition in the gill and sediment.

### Metagenome sequencing

Genomic DNA intended for amplicon sequencing and the newly extracted DNA were both used for metagenomic sequencing. The newly extracted genomic DNA was obtained from gill and whole tissue using the SDS method. The library was constructed by randomly fragmenting the DNA into ~350-bp reads. Following library construction, sequencing was conducted in paired-end 150-bp mode on an Illumina NovaSeq 6000 platform (Tianjin, China). Simultaneously, the long quality-checked DNA was sent to Novogene (Tianjin, China) for library preparation and sequencing. For Oxford Nanopore Technologies (ONT) sequencing, the library was generated using the SQK-LSK109 kit (Oxford Nanopore Technologies, UK) in accordance with the manufacturer’s guidelines. Long raw reads were generated through basecalling with Guppy version 6.1.7 (*59*).

### Metatranscriptome sequencing

Total RNA was extracted using TRIzol reagent (Invitrogen, CA, USA) with the guidance of the manufacturer’s protocol. RNA integrity and quantity were measured using the Bioanalyzer 5400 system (Agilent Technologies, CA, USA). cDNA was obtained by removing the prokaryotic and eukaryotic rRNA (rRNA of animal, G-bacteria, and plant) from the total RNA using the TIANSeq rRNA Depletion Kit for the construction of a meta-transcriptomic library. The nucleic acid for metagenome and meta-transcriptome sequencing was subjected to NovaSeq 6000 system (Illumina) at Novogene (Tianjin, China) with paired-end mode and a read length of 150 bp, and the ONT library was sequenced on the PromethION platform at Novogene.

### Spatial metabarcoding sequencing

We used the Stereo-seq formalin-fixed paraffin-embedded (FFPE) pipeline (BGI, China) to investigate the spatial distribution pattern between the two 16*S* phylotypes. The gills from six specimens were prefixed overnight with 4% PFA and then embedded in paraffin. Three continuous sections from the embedded block were cut at 10-μm thick. The second section was designated for chip loading to capture rRNA, while the remaining two sections were used in staining (either H&E or FISH). The tissue section was adhered to the Stereo-seq chip (BGI, China) surface and incubated at 37°C for 3 min. The tissue sections were fixed in methanol and incubated at −20°C for 40 min before Stereo-seq library preparation. Where applicable, the same sections were stained with a nucleic acid dye (Thermo Fisher Scientific, Q10212), and imaging was conducted using a Stereo OR 100 microscope in the fluorescein isothiocyanate channel before in situ capture. After washing with 0.1× SSC buffer (Thermo Fisher Scientific, AM9770) supplemented with ribonuclease (RNase) inhibitor (0.05 U/ml; NEB, M0314L), the tissue sections placed on the chip were permeabilized using 0.1% pepsin (Sigma-Aldrich, P7000) in 0.01 M HCl buffer. They were incubated at 37°C for 5 min and subsequently washed again with the same 0.1× SSC buffer with RNase inhibitor. cDNA was synthesized on the chip using the FFPE mix solution, consisting of 158 μl of FFPE RT buffer mix, 30 μl of FFPE RT enzyme mix, 10 μl of FFPE RT oligo, and 2 μl of FFPE dimer at 42°C for 5 hours. The cDNA-containing chips then underwent treatment with the Prepare cDNA Release Mix (cDNA release enzyme and cDNA release buffer) overnight at 55°C. The harvested cDNA was purified using VAHTSTM DNA Clean Beads (0.8×) and subsequently amplified in the amplification solution, which included 42 μl of cDNA, 50 μl of cDNA amplification mix, and 8 μl of FFPE cDNA primer mix. The PCR protocol was as follows: initial denaturation at 95°C for 5 min, followed by 15 cycles of denaturation at 98°C for 20 s, annealing at 58°C for 20 s, and extension at 72°C for 3 min, concluding with a final extension at 72°C for 5 min. After quantification using the Qubit dsDNA HS kit, the cDNA product was used for library construction according to the guidelines of the Stereo-seq 16 Barcode Library Kit V1.0. Raw reads were retrieved in paired-end 75-bp mode using MGI DNBSEQ-T7.

### Spatial metabarcoding analyses

Fastq files were generated from an MGI DNBSEQ-T7 sequencer. Coordinate ID (CID) and molecular ID (MID) are contained in the read 1 (CID: 1 to 25 bp, MID: 26 to 35 bp), while the corresponding read 2 consists of the 16*S* rDNA sequences. The current SAW software version 8.0.2 (*60*) could not differentiate the two 16*S* phylotypes with the difference of a single base pair. To visualize the patterns, the preseparation of reads containing the differentiated base pair fully matching the full-length 16*S* rRNA gene was adopted. In detail, the normal analysis of the Stereo-seq read was completed, with all the raw reads, single-stranded DNA image, mask file, and reference. The STAR genome reference was prepared with the de novo assembled transcriptional profile and the 16*S* rRNA sequence (phylotype A). The produced .*bam* files were processed, with the region (16*S* rRNA sequence: 907 to 953) kept. The phylotype A reads were extracted if they were uniquely mapped and completely matched to 16*S* rRNA (NH:i:1, MAPQ = 255, CIGAR = 47M). Meanwhile, the phylotype G reads were extracted as the same way, with the substitution of a single base pair (A to G). Then, two independent analyses of them were run using the SAW pipeline. The count of mapping reads spatially was shown as the two-dimensional locations and the corresponding mapping number (the tab-delimited file with three columns). Given the cellular size (about 10 μm), these counts were aggregated into bin 20 (20 × 20 DNA NanoBalls). Bin 20 spots were considered valid only if they had at least 180 reads. The ratio of 16*S* phylotype (A or G) was then calculated, with the frequency histogram shown (fig. S7). Compared to other frequencies of phylotypes (every 5%) in spatially resolved bins, the two frequencies (95 to 100% and 0 to 5%) were typically higher, accounting 84.49 ± 10.76%. Therefore, a dominant phylotype was called for bins having a ratio above 95% and represented spatially using Matplotlib in Python.

### Symbiont genome assembly, binning, and annotation

Short raw reads were trimmed using Trimmomatic version 0.39 (*61*) with the following settings: TruSeq3-PE-2.fa:2:30:10:8:true SLIDINGWINDOW:5:20 LEADING:3 TRAILING:3 MINLEN:36. Clean reads were assembled using Megahit version 1.2.9 (*62*) with default settings. The MAGs were binned using MaxBin version 2.2.7 (*63*), with the cutoff of contig length from 1000 to 2000 for the optimization. To reduce the host contamination, we conducted a decontamination process using BlobTools version 1.1.1 (*64*) with default settings, and then sequences belonging to the phylum Pseudomonadota were selected for downstream analyses. To obtain the circular-level genome of the symbiont, first, the ONT long reads were mapped to the highest quality MAGs; second, these long reads mapped were assembled using NextDenovo version 2.5.2 (*65*); last, clean reads from each sample (i.e., Illumina) were input into NextPolish version 1.4.1 (*66*) to polish the circular-level genome. Then, these genomes were evaluated for completeness and contamination using CheckM2 version 0.1.3 (*67*). GTDB-TK version 2.1.1 (*68*) was used to determine the taxonomy of symbionts at the genome level. The matrix of pairwise ANI of 30 MAGs was generated using FastANI version 1.34 (*33*). The Wilcoxon rank-sum test was used to assess differences in ANI values across symbiont phylotypes. 16*S* rRNA genes and open reading frames of MAGs were predicted by Prokka version 1.14.6 (*69*) in the single-genome mode. These predicted genes of the pangenome were searched against the nonredundant (NR) database using BLASTp in DIAMOND version 2.0.15.153 (*70*) with an *E* value cutoff of 1 × 10^−5^. The results were further used for Gene Ontology annotation by Blast2GO version 6.0 (*71*). Meanwhile, Clusters of Orthologous Group 2020 (*72*) was adopted to classify the functional groups of genes in the pangenome. The genes of the pangenome were annotated using BlastKOALA (*73*) by searching against the Kyoto Encyclopedia of Genes and Genomes (KEGG) database (table S10). KEGG-Decoder version 1.3 (*74*) was used to assess pathway completeness, and the resulting data were visualized using the *ComplexHeatmap* package in R (*75*).

### Phylotype decomposition, comparison, and phylogenomic analysis

A pangenome was generated using PanPhlAn version 3.1 (*76*) and the built-in scripts of StrainPanDA (*77*). To decompose the diversity of symbionts at high resolution, StrainPanDA was adopted on the basis of the newly constructed pangenome of 30 MAGs and the full set of clean reads. To differentiate between the two symbiont phylotypes, OrthoFinder version 3.1.0 (*78*) was used to identify the potential phylotype-specific genes. We further manually excluded genes truly absent from the other phylotype’s genome by performing BLAST searches against its genome and amino acid sequences. EggNOG-mapper version 2 (*79*) was used to quickly check the different gene functions. The phylogenetic positions of the two symbiont phylotypes were reconstructed among known chemosynthetic bacteria using 57 published SOB genomes and the *B. azoricus* methane-oxidizing bacteria (MOB) symbiont as an outgroup. The phylogenomic analysis was conducted using VEHoP version 1.0 (*80*) to determine their positions at the phylogenomic level. The phylogenomic tree was visualized using tvBOT (*81*).

### Transcript assembly, annotation, and quantification

The raw reads were filtered using trimmomatic version 0.39 with the settings (TruSeq3-PE-2.fa:2:30:10:8:true SLIDINGWINDOW:5:20 LEADING:3 TRAILING:3 MINLEN:75). Then, qualified reads were mapped to the pangenome using Bowtie version 2.3.5 (*82*), resulting in the symbiont-derived reads and symbiont-free reads. The symbiotic-free reads were subjected to Trinity version 2.13.2 for de novo assembly (*83*). To prepare for spatial metabarcoding analysis, the host transcripts were first purified using BlobTools version 1.1.1 (*64*) to remove contaminated contigs and subsequently assessed for coding potential using TransDecoder version 5.7.1 (*84*). The genes of the pangenome were annotated using BlastKOALA (*73*) by searching against the KEGG database. KEGG-Decoder version 1.3 (*74*) was used to assess pathway completeness, and the resulting data were visualized using the *ComplexHeatmap* package in R (*75*). Salmon version 1.9.0 (*85*) was performed to quantify the gene expression levels of the symbiont.

### ^14^C-bicarbonate incubations and rate calculations

Radiotracer assays were used to determine the DIC assimilation rate by introducing a ^14^C-labeled DIC tracer to homogenized gill tissue and live clam samples, respectively, and quantifying the amount of ^14^C incorporated into particulate organic carbon (POC) (*86*). The experimental design details are as follows (Fig. 5B and fig. S15B):

#### Live clam incubations

Whole live clams (*n* = 56, eight stations) were incubated in 20-ml sterile serum vials following a previously published protocol (*50*). At each station, six experimental samples and one control sample were prepared, and 15 ml of in situ sterile seawater was added to each vial containing a clam. Subsequently, 100 μl of ^14^C-DIC solution [~3.7 × 10^4^ becquerel (Bq)] was injected into each vial. Control samples were terminated by adding formaldehyde to a final concentration of 4% to arrest biological activity. All vials were inverted gently to mix and incubated for ~12 hours at in situ temperature. After incubation, CO_2_ fixation in the experimental vials was stopped by adding formaldehyde to the same final concentrations as in the controls. The seawater incubation medium was first filtered onto a 0.22-μm GSWP filter membrane (polyethersulfone, Millipore) and rinsed three times with 35 ‰ sodium chloride (NaCl) solution. The clam was then removed, ground thoroughly using a tissue grinder, and filtered onto the same 0.22-μm membrane, followed by three additional rinses with 35 ‰ NaCl solution. The filter was placed in a 7-ml scintillation vial containing a 6 ml of scintillation cocktail (Ultima Gold Cocktail, PerkinElmer), and the radioactivity was measured with a liquid scintillation counter (Tri-Carb 3100TR, PerkinElmer) (*87*).

The turnover rate constant (*k_n_*) of DIC was calculated using Eq. 1, and DIC assimilation rate (*Assim*-*rate*) was estimated using Eq. 2

$$k_{n}=-\text{ln}\;\frac{\frac{1-\left(\text{DPM}{-}^{14}C-\text{POC}\right)}{\text{DPM}{-}^{14}C-\text{DIC}}}{t}$$ (1)

$$\text{Assim-rate}=k_{n}\times \left[\text{DIC}\right]*0.015*10^{-6}$$ (2)

where *k*_n_ is turnover rate constant (day^−1^), *Assim-rate* is DIC assimilation rate (nmol C·clam^−1^·day^−1^), DPM (disintegrations per minute) is a unit that quantifies the rate at which radioactive atoms decay, *DPM-^*14*^C-POC* is radioactivity measured on the filter, *DPM-^*14*^C-DIC* is the total activity of the added DIC tracer, *t* is the incubation time (day), [*DIC*] is the in situ DIC concentration in bottom seawater (mmol·liter^−1^), and 0.015 is the incubation volume of a single live clam (0.015 liters).

#### Homogenized gill tissue incubations

Gill tissues (*n* = 16, four stations) dissected from fresh samples were homogenized and immediately stored in 20% glycerol in sterile seawater and then slowly thawed on ice before downstream analyses. The experimental design was as follows: (i) to minimize the impact of varying symbiont numbers on our results, we collected samples from four stations, and four individuals per station; (ii) to create a uniform starting point, samples from each station were mixed, and the resulting mixture was divided into 16 replicates, and these replicates were then assigned to one of four incubation groups at different temperatures (5°, 12°, 20°, and 28 °C) and incubated in the dark for 46 to 47 hours; (iii) within each temperature group, we prepared four replicates of gill tissue samples: three experimental samples and one negative control. These samples were placed into 10-ml serum vials, ensuring no headspace, and sealed with sterile PTFE septa and aluminum caps. After that, 100 μl of ^14^C-DIC solution (~4.1 × 10^4^ Bq) was injected into each serum vial through the stopper by displacing the same volume of water. Before injecting the ^14^C-DIC tracer, the microorganisms in the controls were killed by adding 0.5 ml of 100% trichloroacetic acid. After incubation, microbial activity was terminated using the same procedure as for controls, and the suspensions were filtered onto 0.2-μm GSWP membranes. Filters were rinsed with 35 ‰ NaCl solution (*88*) and transferred into 7-ml scintillation vials containing 6-ml scintillation cocktail. The radioactivity of the filters was determined using a liquid scintillation counter (*87*). The turnover rate constant (*k*_n_) and assimilation rate were calculated as above. The DIC concentration was measured using the 0.22-μm-filtered incubation seawater. As mentioned before, the symbiont abundance in each vial represented 25% of that in a whole clam. The DIC assimilation rate (*Assim-rate*) of the homogenized gill tissue was calculated as in Eq. 3, which includes a multiplication by 4 to obtain a normalized unit of nmol C (nmol C·clam^−1^·day^−1^)

$$\text{Assim-rate}\;=k_{n}\times \left[\text{DIC}\right]*0.010*10^{-6}*4$$ (3)

where *Assim-rate* is the DIC assimilation rate (nmol C·clam^−1^·day^−1^), [*DIC*] is 3.07 [DIC concentration in incubation seawater (mmol·liter^−1^)], and 0.010 is the incubation volume of a single live clam (0.010 liters).

## Supplementary Materials

### The PDF file includes:

Supplementary Text Figs. S1 to S17 Legends for tables S1 to S11 Legend for supplementary commands References

### Other Supplementary Material for this manuscript includes the following:

Tables S1 to S11

Supplementary commands
