# Supplementary material for: Intricate chemosymbiosis in a widespread shallow-water thyasirid clam
Source: Sci Adv. 2026 Mar 4;12(10):eadw8163. doi: 10.1126/sciadv.adw8163 (PMC12959385; doi:10.1126/sciadv.adw8163)
Supplement: Supplementary file 1 — Supplementary Text Figs. S1 to S17 Legends for tables S1 to S11 Legend for supplementary commands References [file sciadv.adw8163_sm.pdf]

Science Advances
AAAS

Supplementary Materials for

Intricate chemosymbiosis in a widespread shallow-water thyasirid clam

Menggong Li *et al.*

Corresponding author: Jin Sun, jin_sun@ouc.edu.cn; Guang-Chao Zhuang, zgc@ouc.edu.cn

Sci. Adv. **12**, eadw8163 (2026)
DOI: 10.1126/sciadv.adw8163

The PDF file includes:

Supplementary Text
Figs. S1 to S17
Legends for tables S1 to S11
Legend for supplementary commands
References

Other Supplementary Material for this manuscript includes the following:

Tables S1 to S11
Supplementary commands

Supplementary Text: Rough carbon flux estimation for *Thyasira tokunagai*

Materials and Methods

Sampling description

In detail, 1) samples for genomic DNA extraction were stored in 70% ethanol or frozen at -80 °C; 2) samples for fluorescence *in situ* hybridization (FISH) were initially fixed in 4% paraformaldehyde (PFA) at 4 °C overnight and then transferred to 100% methanol at -20 °C; 3) samples for RNA extraction were preserved at RNA stabilization solution (Thermo Fisher Scientific Inc., Waltham, USA) at 4 °C overnight and long-term preserved at -80 °C; 4) bottom seawater samples for radioactive isotope tracing experiment were stored in the dark at 4 °C; 5) gill tissue for radioactive isotope tracing experiment were freshly dissected, homogenized in 60% sterile glycerol solution, and flash-frozen at -80 °C; 6) samples for transmission electron microscope (TEM) were stored in the mixed solution (2.5 % glutaraldehyde in phosphate buffer and 2% PFA) at 4 °C; and 7) tissue samples for stable isotopic analysis were removed from the shell and then kept at -20 °C; 8) sediment samples from two stations (NYS1 and NYS3) were collected and immediately frozen at -20 °C for the environmental microbial community analysis; 9) the sediment cores were immediately placed vertically on a board (see **fig. S10A**) for the measurement of sediment oxygen and the enumeration of clams in each layer.

Sediment oxygen measurement

In the July 2025 cruise, we collected the sediment core sample from two stations (i.e. 2507_N04 and 2507_N16) to explore the oxygen penetration depth and the distribution of *T. tokunagai*. Sediment oxygen was measured in duplicates or triplicates within 2 h after box corer sampling, using an oxygen microelectrode (OX-25, Unisense, Denmark) installed on a micromanipulator with a resolution of 100 µm.

Mitochondrial genome assembly and annotation

Raw reads were trimmed to remove low-quality sequences and adapters using Trimmomatic v.0.39 (61) with the following parameters: ILLUMINACLIP: TruSeq3-PE-2.fa:2:30:10,

LEADING:20, TRAILING:20, SLIDINGWINDOW:4:15, MINLEN:100. NOVOPlasty v.4.3.1 (89) were employed to construct the mitochondrial genomes with default settings (160M randomly selected reads per sample). Assembled mitochondrial genomes were annotated on the MITOS web server (90) with the default setting except "the genetic code: 5 invertebrates".

Host phylogenetic and population structure analysis

A total of 24 *cox1* gene sequences in the family Thyasiridae were downloaded from the NCBI, and three lucinid sequences served as the outgroup in the phylogenetic analysis **(table S11)**. Then, these sequences were aligned using MUSCLE v.5.1 (91), and the ambiguous alignment was trimmed using Gblocks v.0.91b (92). The phylogenetic tree was constructed using the Maximum Likelihood (ML) method in MEGA-X v.10.2.2 (93) and 100 bootstraps, and the HKY + Γ model was suggested as the best model based on both AIC- and BIC-based methods. To understand the population differentiation of *T. tokunagai* in the Yellow Sea, the DnaSP v.6 (94) and PopART v.1.7 (95) with the median-joining Network method were employed to investigate the haplotype network based on the alignment file. Furthermore, 13 protein-coding genes of 30 individuals were aligned separately using MAFFT v.7.515 (96) with the default parameter. Population structure analysis was performed based on the mitochondrial genome by STRUCTURE v.2.3.4 (97) with the settings of "*K*: from 2 to 7, 2,000,000 iterations, and 10% of burnin". The most optimal *K* was determined using Structure Harvester (98) web server with the delta *K* method.

Phylogenetic analysis of symbiont based on 16S rRNA gene

A total of sixteen 16S rRNA gene sequences were downloaded, and the 16S rRNA gene tree of symbiont was constructed with the *Escherichia coli* 16S rRNA gene as the outgroup. The alignment analysis and phylogenetic method were referred to as the *cox1* gene analysis. The best model was the HKY +Γ +I model and 100 bootstrap was also used to evaluate the confidence level.

^{14}C-bicarbonate incubations of bottom seawater

Bottom water from the same eight stations used for live clam incubations was collected (n = 32, 8 stations) to measure the DIC assimilation rates. At each station, three experimental samples and one negative control were prepared. Samples were placed into 20 mL serum vials without headspace and sealed with sterile PTFE septa and aluminum caps. Subsequently, 100 μL of ^{14}C-DIC solution (~ 3.7×10^4 Bq) was injected through the septa into each vial, replacing an equivalent volume of seawater. Before ^{14}C-DIC tracer addition, controls were fixed with 0.5 mL 100% trichloroacetic acid. All samples were incubated for 24 hours at *in situ* temperature and were terminated by adding trichloroacetic acid after incubation. The killed samples were filtered onto 0.2 μm GSWP membranes, rinsed with 35‰ NaCl solution and radioactivity was determined using the liquid scintillation counter (87). The turnover rate constant (k_n) was calculated as described above (the equation No. 1). The DIC assimilation rate (*Ass-rate*) of seawater was normalized to incubation volume, with unit of nmol C·100 mL^{-1}·day^{-1}

A rough carbon flux estimation of *Thyasira tokunagai*

We acknowledge that there is substantial uncertainty in our estimation of the spatial distribution of *T. tokunagai* across the Yellow Sea. This uncertainty stems from multiple interconnected limitations: first, the reliance on limited occurrence records coupled with the inherent small scale patchy distribution of infaunal organisms—factors that likely led to the overestimation of this species' specific biological carbon sink contribution; second, our failure to detect any relationships between *T. tokunagai* density and key physical parameters (bottom layer temperature, depth, and dissolved oxygen). Without such correlations, machine learning models (which rely on meaningful environmental predictors to capture species-environment associations) could not be effectively constructed, especially given the large spatial scale of the Yellow Sea and the paucity of available data. The total region for flux estimation was restricted by the maximum region from 162 sampling stations (**Table S1 and fig S17**). We therefore provide a rough estimation of carbon flux for *T. tokunagai*, with the corresponding method described below.

The Python package pykrige.ok version 1.7.2 (99) was employed for the kriging interpolation

among stations, predicting the spatial pattern of species density with a resolution of 0.005 degrees (division unit) and under the parameters (spherical model, 'nugget': 0.1, 'range': 1.0, 'sill': 0.5). The estimation of total annual carbon fixation flux in the Yellow Sea contributed by this *T. tokunagai* holobiont (i.e. live clam) followed the following procedure: we measured the DIC assimilation rate of live thyasirids from 8 stations (6 replicates in each station, under the in-situ temperature and DIC levels) and then obtained the average rate for the following flux estimation. Annual carbon fixation flux (*Ann-Flux*) in each station was calculated using the equation No. (4), mainly incorporating the interpolated *T. tokunagai* density. The total annual carbon fixation flux is the sum of each division unit.

$$\boldsymbol{Ann - Flux = \sum_{n=1}^{area} An \cdot 29.3 \cdot 12 \cdot 365 \cdot 10^{-9}} \quad \mathbf{(4)}$$

Ann-Flux: annual carbon fixation flux (g C·yr $^{-1}$).

A_n: the predicted spatial abundance of *Thyasira tokunagai* via kriging exploitation (clam·m^{-2}, **fig S17**).

29.3: the average level of DIC asslimilation rate (nmol·clam^{-1}·day^{-1}).

12: the molar mass of carbon (g·mol^{-1}).

365: assuming the total number of days in a year (day).

Biological distribution data of *Thyasira gouldii* complex (including *T. tokunagai* and *T. gouldii*) were retrieved from the Ocean Biodiversity Information System (OBIS; https://obis.org/) and the NBN Atlas (https://nbnatlas.org/).

Results and Discussion

Clone library construction (Supplementary Note 1)

To ensure that the difference of one nucleotide between the two oligotypes is not caused by sequencing error, the DNA of two representative samples (i.e., “NYS3_4” and “NYS3_5”) was used to construct the clone library. Firstly, to obtain the gene target fragment, the full-length 16S rRNA genes were amplified following the protocol in the main text, and the PCR product was further purified by using the E.Z.N.A. Cycle Pure Kit (Omega Bio-tek, Inc). Then, a total of 70 ng target fragments were inserted into the vectors. All experimental procedures were followed by pClone007 Blunt Simple Vector Kit (Tsingke, China) with standard protocol. A

total of 23 valid single clones were selected randomly for Sanger sequencing by BGI (Qingdao, China) from both ends. Raw reads of full-length 16S rRNA genes were assembled to a consensus sequence with the primers removed. Collectively, a total of 23 nearly full-length monoclonal sequences were obtained: 11 *Sedimenticola* sp. (ex *Thyasira tokunagai*) strain G, 1 strain of *Spirochaeta*_2 genus from the NYS3_4 sample, 8 *Sedimenticola* sp. (ex *Thyasira tokunagai*) strain G, and 3 strains of the genus *Spirochaeta*_2 from the NYS3_5 sample.

A rough carbon flux estimation of thyasirid holobionts and their potential role in carbon cycling (Supplementary Note 2)

Thyasirids burrow deeply into the suboxic zone of the Yellow Sea and may store fixed carbon in sediments (25), with moderate turbulence here facilitating organic carbon retention compared to the waves of intertidal zones. While hydrodynamic regimes influence sediment carbon storage, sediment organic matter composition and microbial metabolic activity also act as potential factors (100).

The estimation of carbon fixation flux are rough estimates and subject to further revision. In details, we applied kriging interpolation to predict the spatial distribution of *T. tokunagai* across the Yellow Sea based on 162 sampling records from nine cruises. There were three high-density aggregations of thyasirids in cold water mass regions in the Yellow Sea (**fig. S17**), which might be the major contributors of carbon assimilations. Given the estimated 0.128 mg C assimilated by a single thyasirid per year (quantified in live clam experiments), the peak fixation rate could be up to 0.18 g $C \cdot m^{-2} \cdot yr^{-1}$ occurring in the cold-water mass region of the northern Yellow Sea. Considering the areal extent of the Yellow Sea, we estimated that the total annual carbon fixation rate by *T. tokunagai* holobionts in the region was up to approximately 2.74 Gg $C \cdot yr^{-1}$, based on the estimated thyasirid population abundance and the live clam fixation capacity, equal to 1.3‰ in the Chinese coastal blue carbon ecosystems (up to 2.06 Tg $C \cdot yr^{-1}$) (101), suggesting thyasirids from the Yellow Sea as a potential carbon sink. Nonetheless, the magnitude we proposed might be overestimated since the distribution of infauna thyasirids is patchy, though we observed up to 200 specimens from a 0.1 m^2 box corer. More detailed understanding of the distribution patterns of thyasirids in the Yellow Sea is a critical factor in

precisely quantifying their contributions. Further investigation of the widely distributed *T. gouldii* complex or other endemic chemosymbiotic holobionts is needed to deepen our understanding of the carbon flux contributed from chemosymbiosis.

Fig. S1.

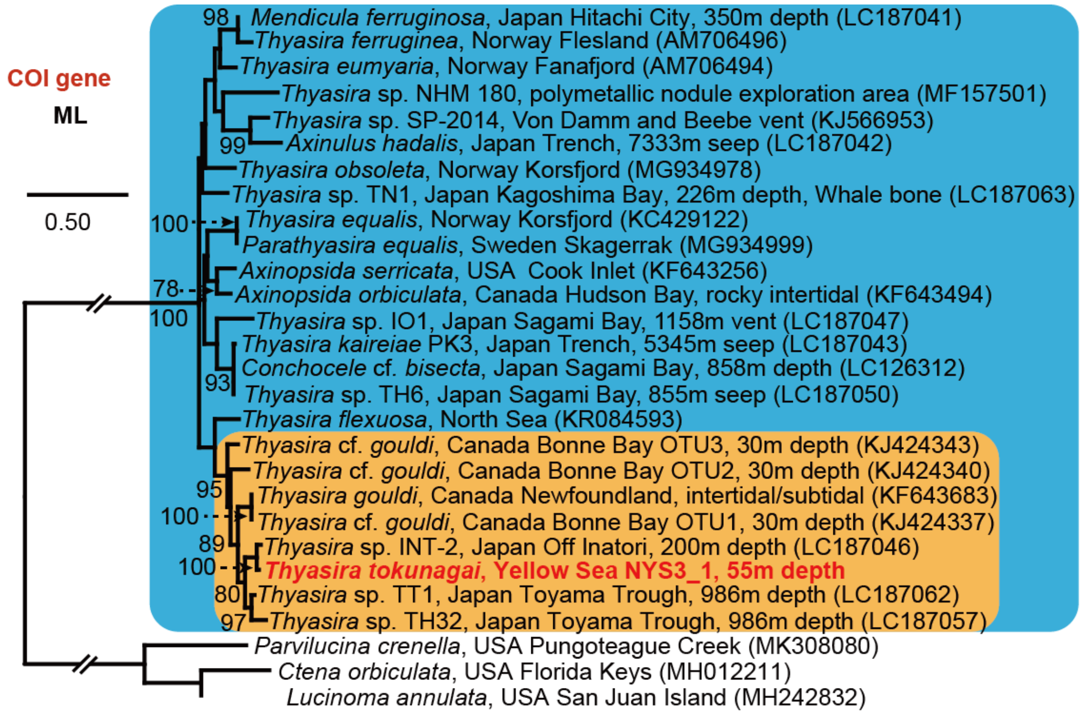

Supplementary Figure S1: Host *cox1* gene tree. Maximum-likelihood tree based on the partial *cox1* gene sequence of *T. tokunagai*. The representative *cox1* sequence of *T. tokunagai* is highlighted in red. This tree was constructed using the HKY +Γ model with 100 bootstraps. The scale bar indicates 0.50 substitutions per site. Values below 50 are hidden.

Fig. S2.

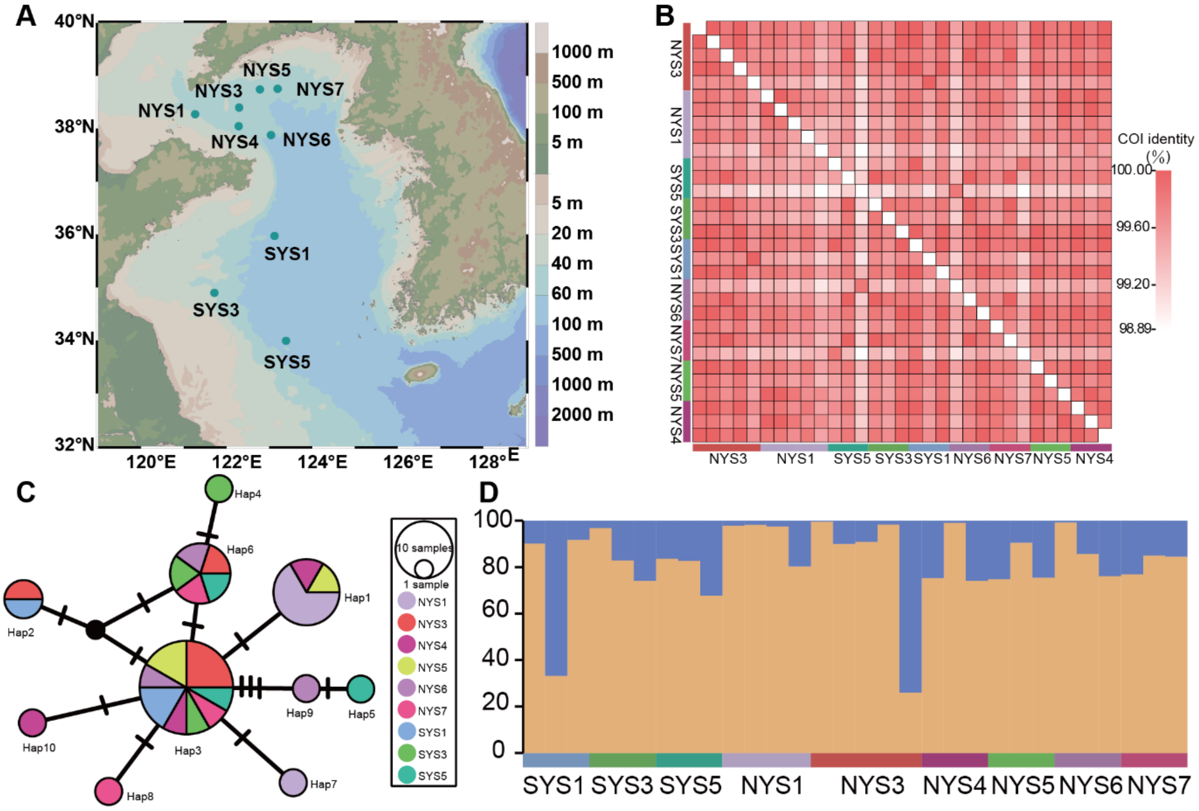

Supplementary Figure S2: Host population analysis. (**A**) Geographic distribution of host population structure sampling stations. (**B**) The pairwise similarity was performed based on the partial *cox1* gene sequences of *T. tokunagai* from 31 individuals located at a total of nine stations in the Yellow Sea. (**C**) The haplotype network of the *cox1* gene from 31 individuals with the median-joining network method had a starburst pattern, indicating no population differentiation. (**D**) Host population structure analysis based on the 13 protein coding genes of the mitochondrial genome of 30 individuals from nine sampling stations in the Yellow Sea. The optimal delta (*K*) value of 2 was used in the host population structure analysis.

Fig. S3.

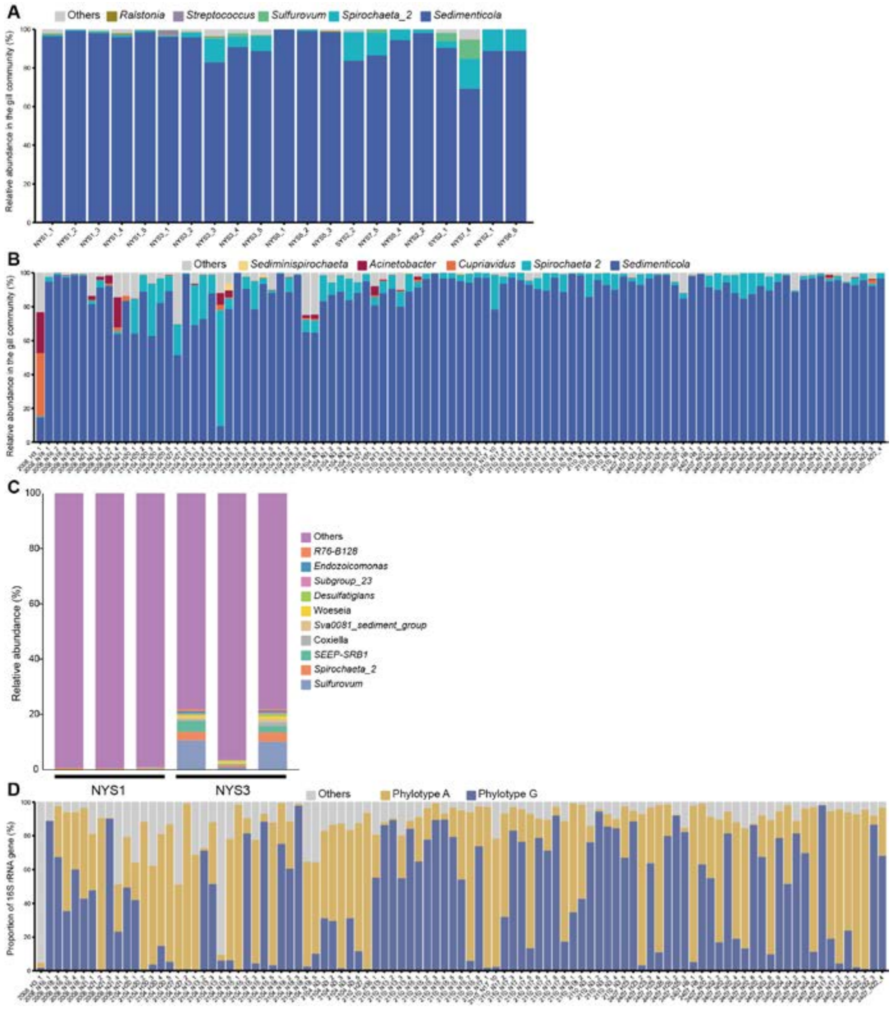

Supplementary Figure S3: Gill and sediment bacterial community composition. (**A**) Genus-level bacterial community composition of gill tissue samples based on the full length and (**B**) V3-V4 region of 16S rRNA gene. The results showed that the symbiont community was dominated by bacteria belonging to the *Sedimenticola* genus. The five most abundant genera are displayed, with all other genera grouped as 'Others'. (**C**) Genus-level bacterial community composition of six sediment samples based on the full length 16S rRNA gene sequences. The ten most abundant genera are displayed, with all other genera grouped as 'Others'. (**D**) Two dominant phylotypes belonging to the genus *Sedimenticola* were identified in gill tissue bacterial communities which we call *Sedimenticola* sp. (ex *Thyasira tokunagai*) ‘phylotype A’ and ‘phylotype G’ based on the V3-V4 region 16S rRNA gene amplicon sequencing. The two symbiont phylotypes are displayed, with all other ASVs grouped as 'Others'.

Fig. S4.

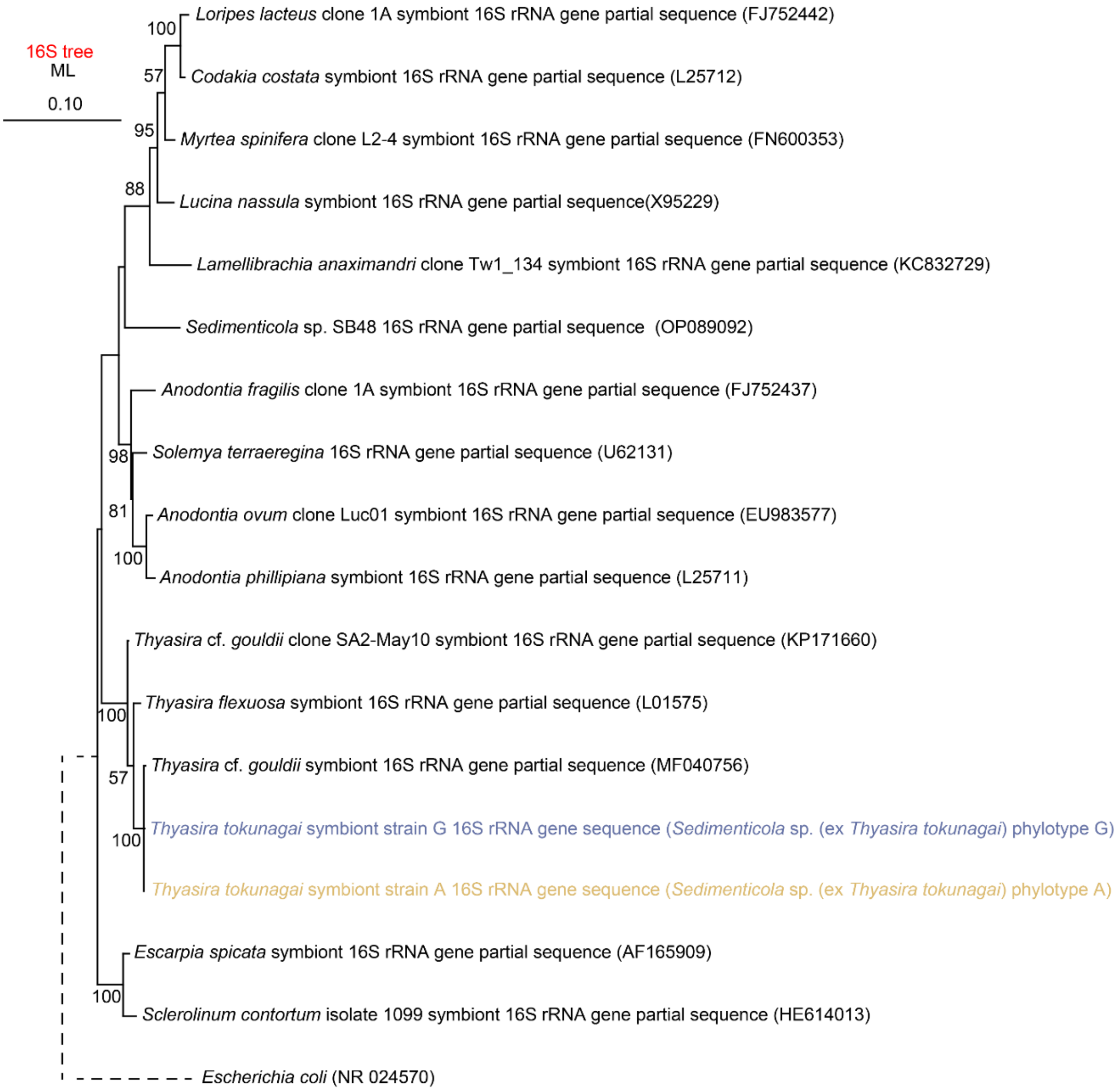

Supplementary Figure S4: Symbiont 16S rRNA gene tree. Phylogenetic analysis based on the full length 16S rRNA gene of symbiont confirmed the close relatedness *Thyasira* cf. *gouldii* symbionts. This tree was constructed based on HKY +Γ +I model with 100 bootstrap values. The scale bar indicates 0.10 substitutions per site. Values below 50 are hidden.

Fig. S5.

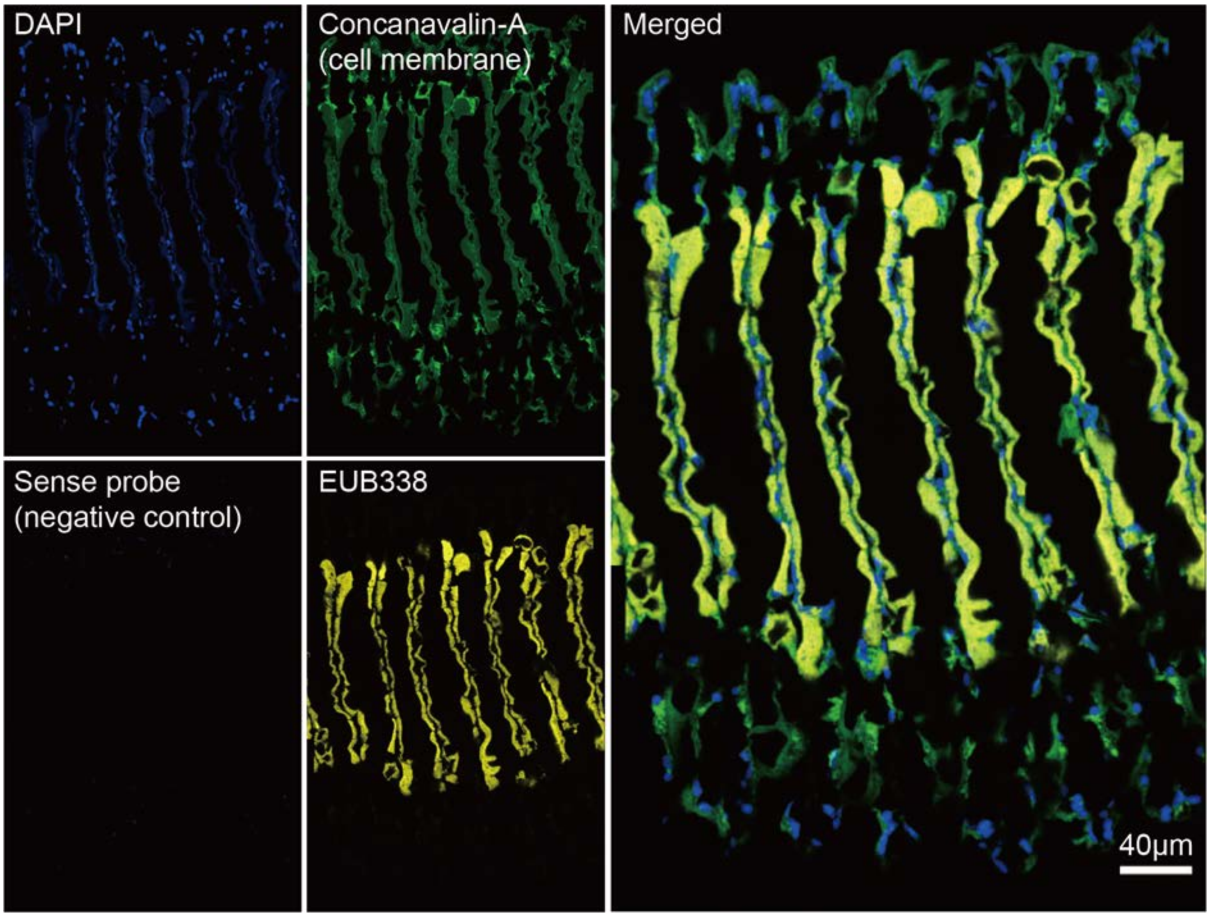

Supplementary Figure S5: Fluorescence *in situ* hybridization (FISH). Fluorescence *in situ* hybridization of gill tissue using the symbiont sense probe as negative control. Cell nuclei were stained with DAPI, the cell membrane was stained by concanavalin-A, and the bacteria were hybridized with a sense probe with CY3-label and a universal probe of EUB338 with CY5-label. The negative control is the reverse complement of the probe sequence, named the sense probe, and the EUB338, as the positive control is a universal bacterial probe.

Fig. S6.

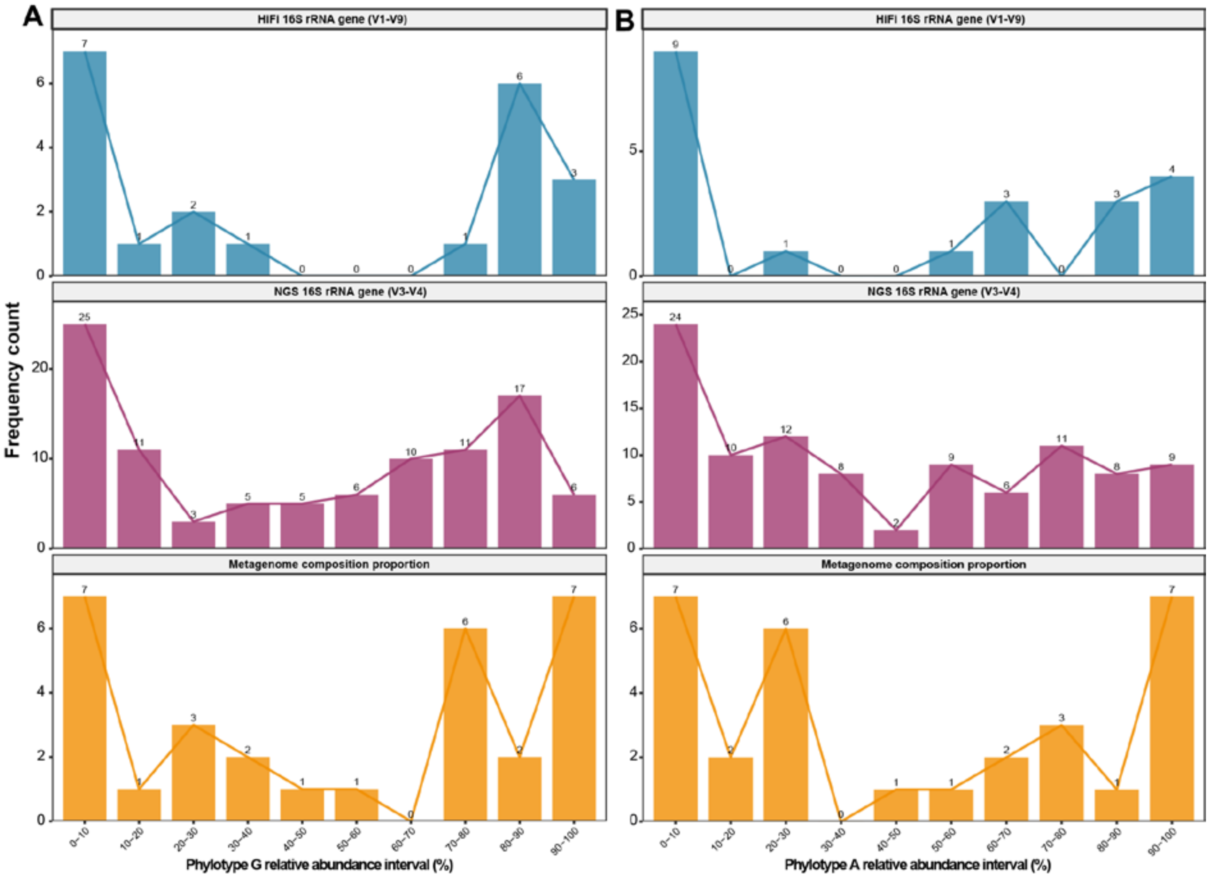

Supplementary Figure S6: Frequency of relative abundance distribution of two phylotypes in 16S rRNA gene and metagenomic datasets. (**A**) Corresponds to symbiont Phylotype G; (**B**) corresponds to symbiont Phylotype A. Each panel includes three subplots: 1) Top subplot (HIFI 16S rRNA gene [V1-V9]): Count of 16S rRNA gene sequences (amplified from V1-V9 region) of the target phylotype, obtained via HIFI sequencing, across different relative abundance intervals. 2) Middle subplot (NGS 16S rRNA gene [V3-V4]): Frequency count of 16S rRNA gene sequences (amplified from V3-V4 region) of the target phylotype, obtained via NGS sequencing, across the same relative abundance intervals. 3) Bottom subplot (Metagenome composition proportion): Proportion of the target phylotype in metagenomic datasets, mapped to the corresponding relative abundance intervals.

Fig. S7.

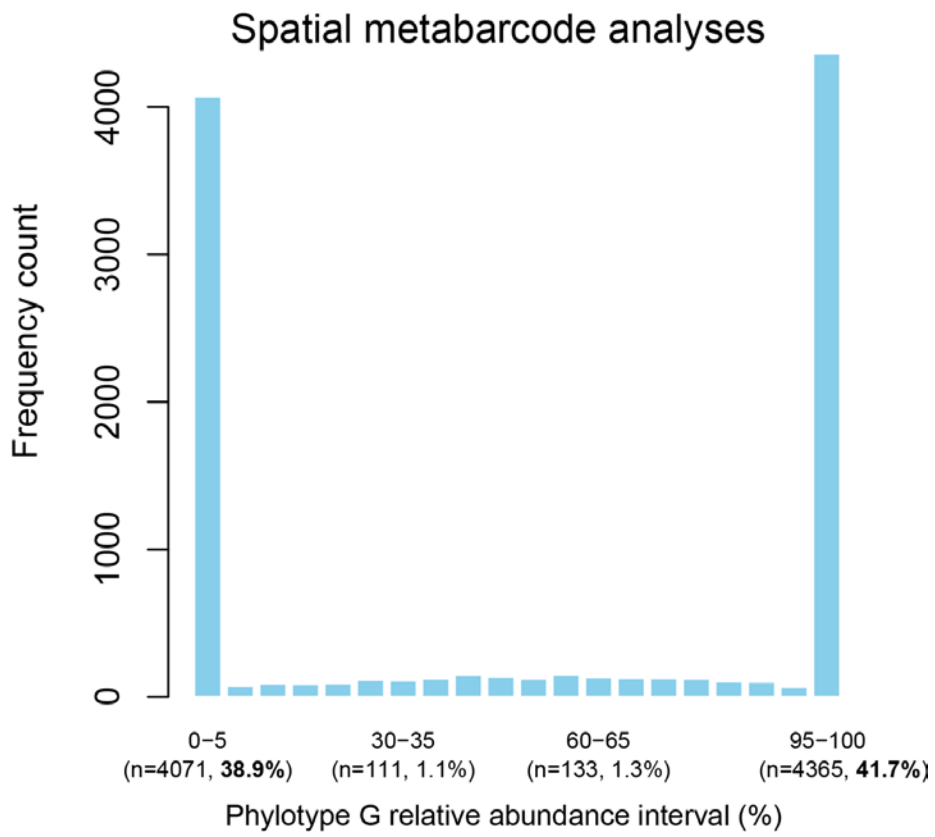

Supplementary Figure S7: Frequency of Spatial metabarcoding of 16S phylotype G ratio. Frequency distribution of the phylotype G ratios within individual bin 20s (10 x 10 µm area) across the six gills examined, indicating that most were dominated by one phylotype (A or G).

Fig. S8.

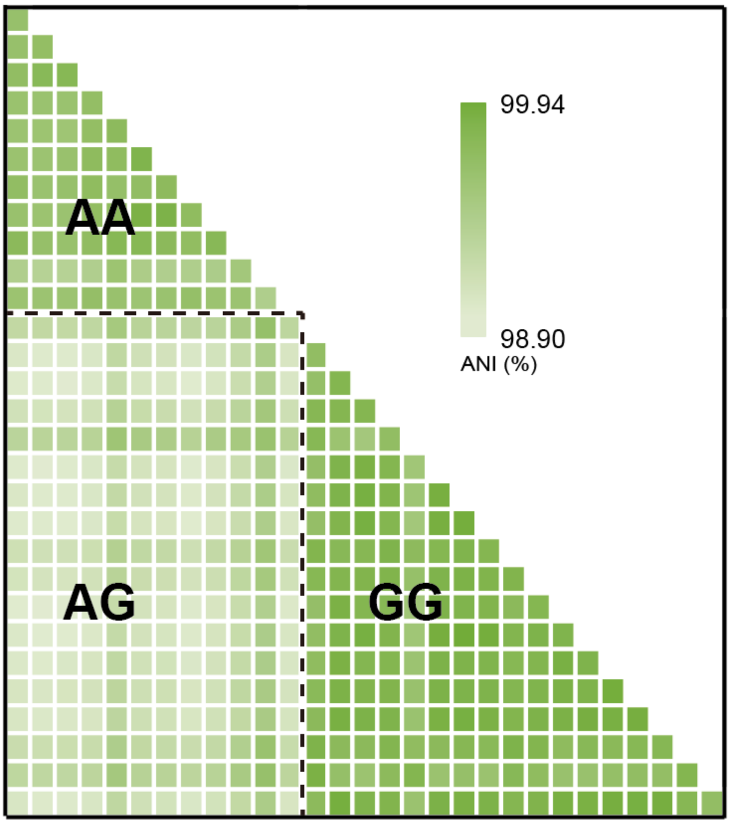

Supplementary Figure S8: ANI matrix of thirty symbiont genomes. The results showed that we could detect genomic differences (*P* = 0.001, Wilcoxon rank sum test) between the symbiont populations. Here, each host was treated as a symbiont population. "A" represented the sample was dominated by phylotype A, and "AG" represented the pairwise average nucleotide identity (ANI) value between two symbiont populations. Other labels followed the same convention.

Fig. S9.

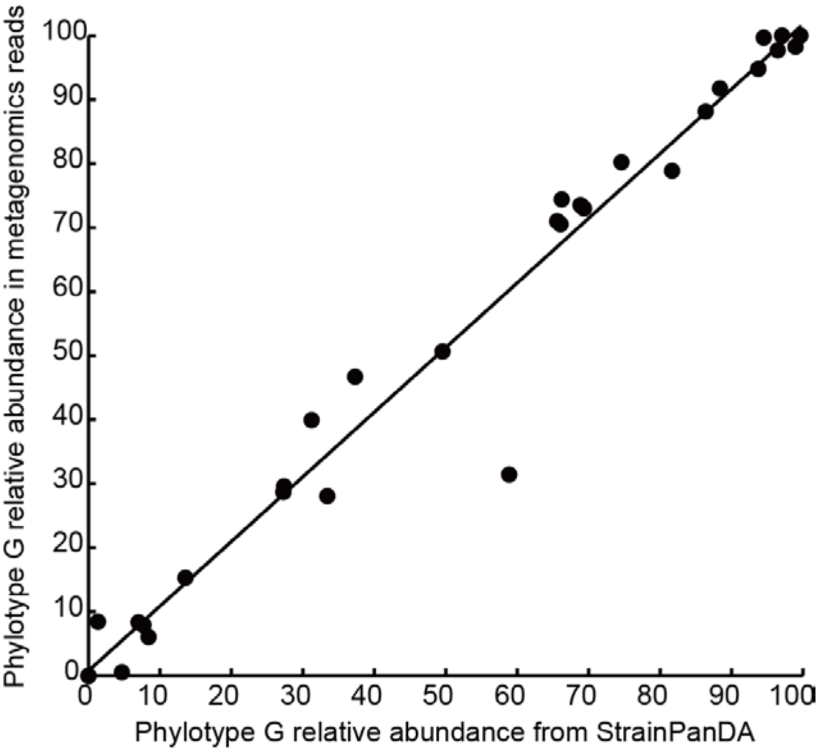

Supplementary Figure S9: Correlation between phylotype G abundance and strain decomposition. There was a positive correlation between the ratio of the base G (at 590th bp of 16S rDNA gene) in the metagenomic reads and the abundance of G strain (the base G presented at 590th bp of 16S rDNA gene) inferred via StrainPanDA ($y = 1.01x + 0.775$, $R^2 = 0.97$).

Fig. S10.

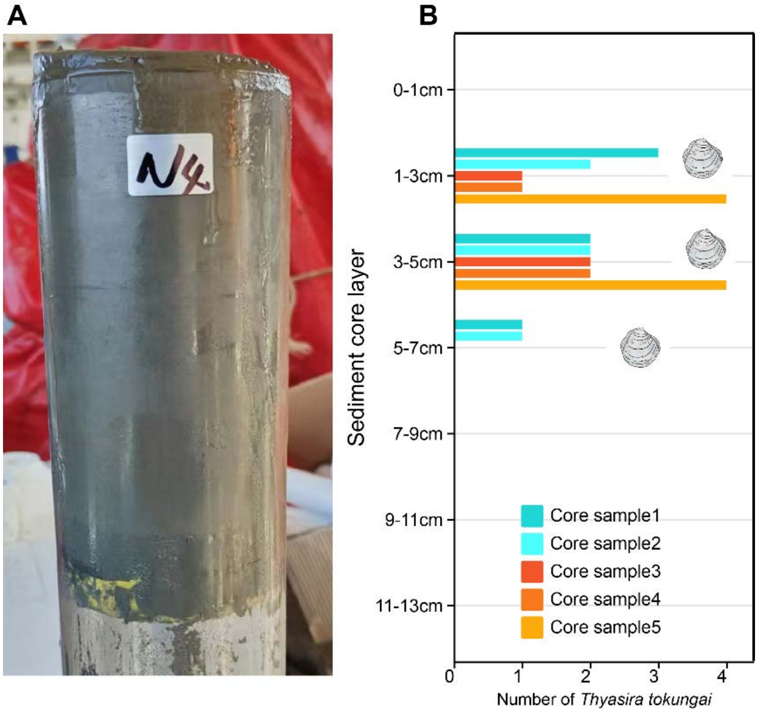

Supplementary Figure S10: the vertical distribution of *Thyasira tokunagai* . (**A**) Onboard preparation of an *in-situ* sediment core sample (10 cm diameter). (**B**) Vertical distribution profiles of *Thyasira tokunagai*. Data were derived from five sediment core samples collected from two stations: 2507_N04 and 2507_N16. Dissolved oxygen penetration depths were 3.2 ± 0.4 mm at station 2507_N04 and 4.7 ± 0.2 mm at station 2507_N16.

Fig. S11.

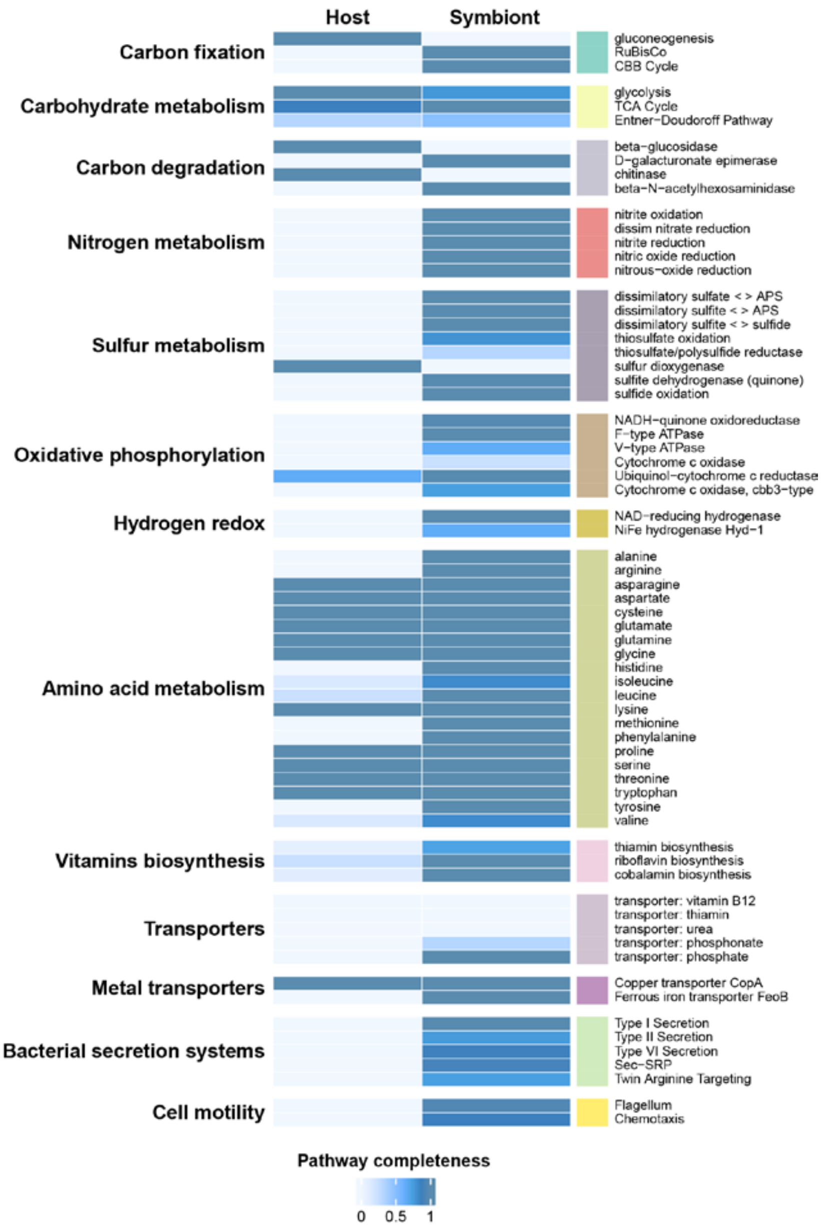

Supplementary Figure S11: Potential metabolic pathways of host and symbiont. This heatmap depicts the predicted pathway completeness of metabolic processes (e.g., carbon fixation, sulfur metabolism) in the host and its symbiont, and colored labels on the right correspond to specific functional modules under each category, with cell shade reflecting the completeness level of the corresponding host/symbiont pathway.

Fig. S12.

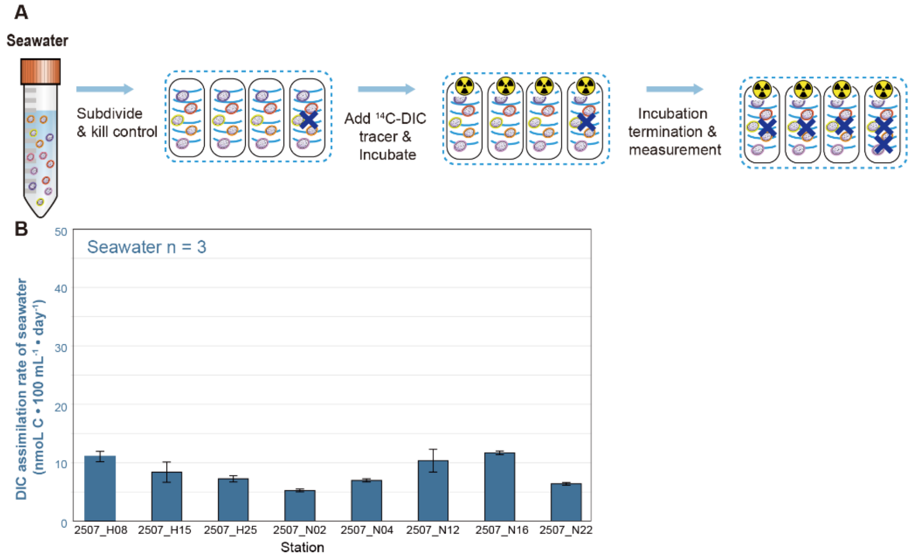

Supplementary Figure S12: Measurement of DIC assimilation rate in seawater. (**A**) Schematic of the ^{14}C-labeled DIC assimilation assay method. To assess the DIC assimilation rate, ^{14}C-labeled DIC tracer was added to seawater samples, and the samples were incubated at simulated in situ temperatures. The 'X' symbol for seawater represents the samples killed with trichloroacetic acid. (**B**) DIC assimilation rates of bottom seawater (n = 3). Seawater assimilation rates are expressed as nanomoles of carbon assimilated per 100 mL of seawater per day.

Fig. S13.

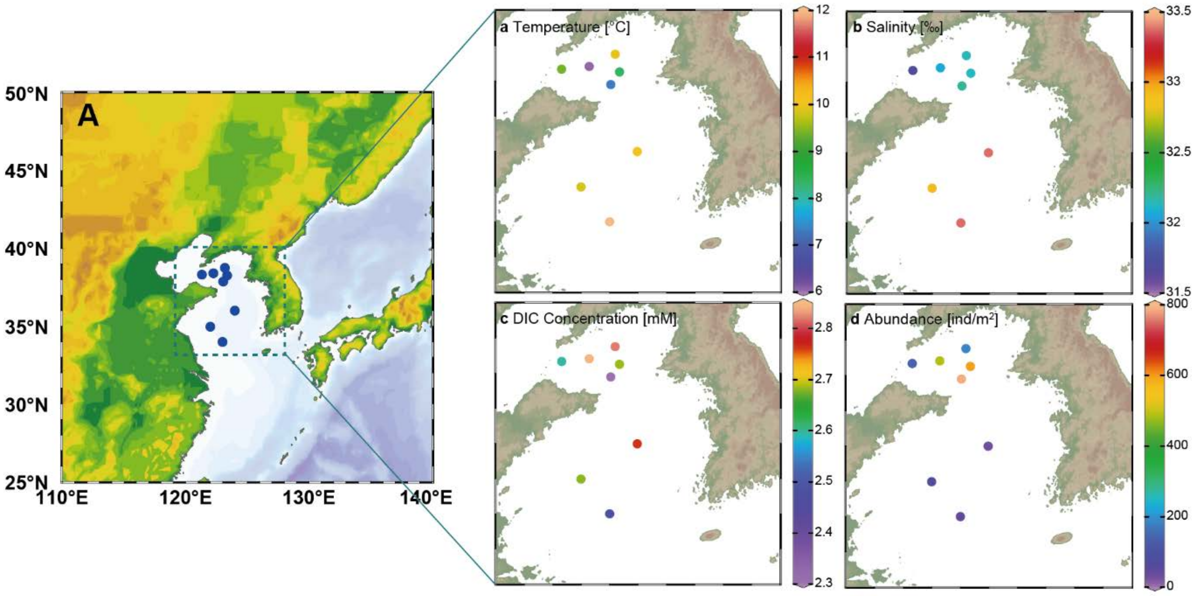

Supplementary Figure S13: *In situ* environmental parameters. (**A**) Distribution of *Thyasira tokunagai* used for DIC assimilation experiment in the Yellow Sea. Data on bottom seawater temperature (**a**), salinity (**b**), DIC concentration (**c**) and clam abundance (**d**).

Fig. S14.

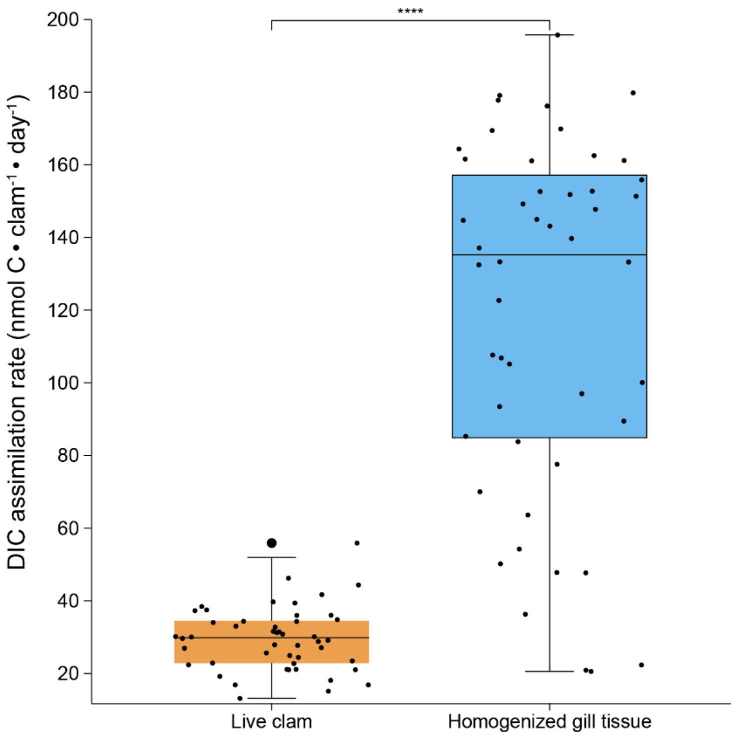

Supplementary Figure S14: Comparison of DIC assimilation rate between live clam and homogenized gill tissue. The top and bottom edges of the box correspond to the upper (Q3) and lower (Q1) quartiles of the group's DIC assimilation rates, while the horizontal line inside the box denotes the group's median value, and the whiskers extending from the box encompass the primary range of extreme values for the group. The asterisk indicates the significance level (****, $P < 0.0001$, Wilcoxon signed-rank test).

Fig. S15.

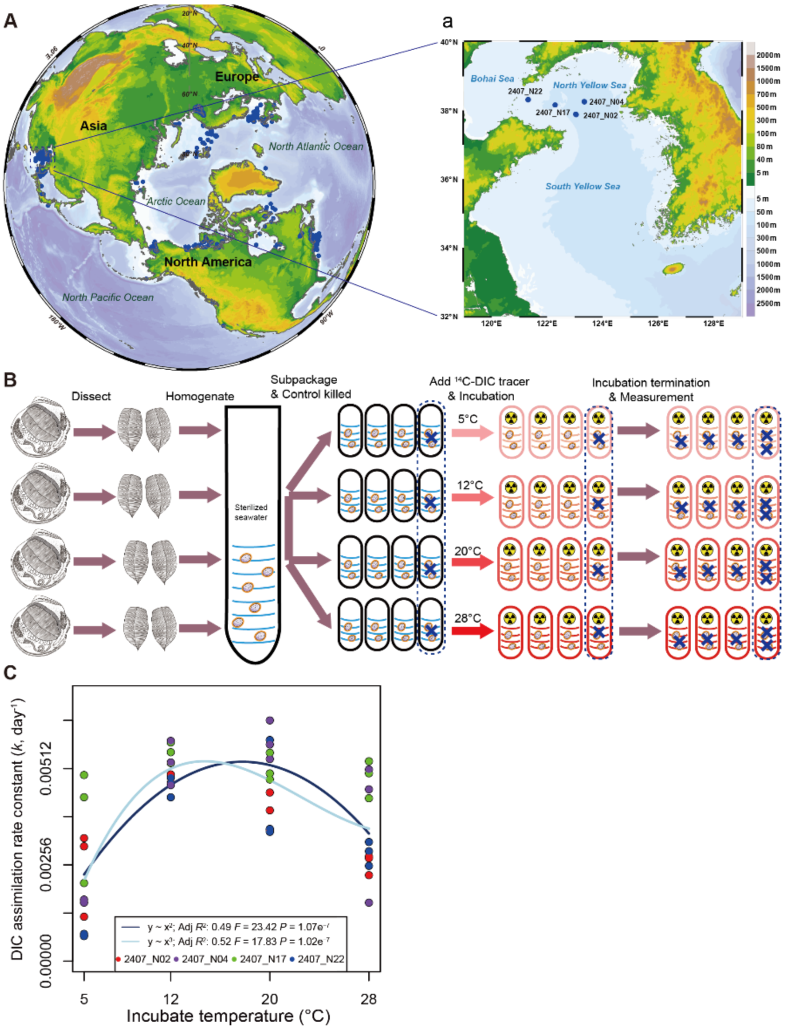

Supplementary Figure S15: Global distribution of *Thyasira gouldii* complex and the measurement of DIC assimilation rate. (**A**) Global distribution of *Thyasira gouldii* complex (including *T. gouldii* and *T. tokunagai*), and (**a**) the distribution of *Thyasira tokunagai* (homogenized gill tissue) used in the DIC assimilation experiment in the Yellow Sea. (**B**) Schematic of the ^{14}C-labeled DIC assimilation assay method. To assess the DIC assimilation rate, ^{14}C-labeled DIC tracer was added to homogenized gill tissue samples, and the samples were incubated at different temperatures (4, 12, 20, and 28 °C). The blue 'X' symbol represents the samples treated with trichloroacetic acid. (**C**) DIC assimilation rate constants (*k*, day^{-1}) at different temperatures (5, 12, 20, and 28 °C) fitted using a binomial equation and trinomial equation.

Fig. S16.

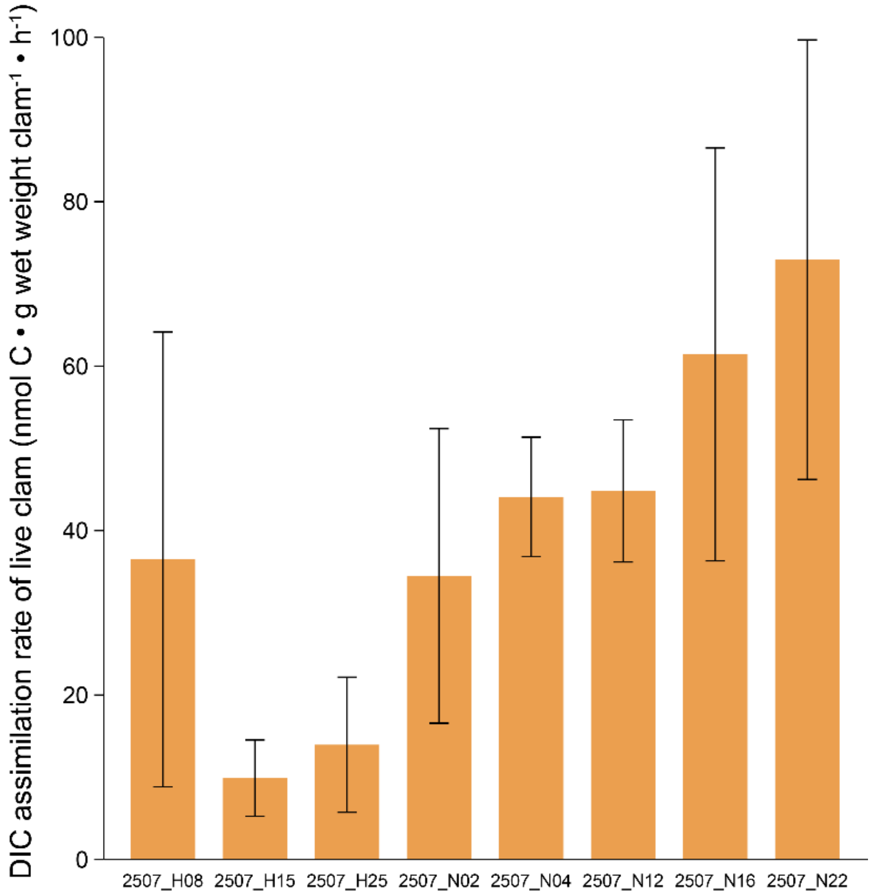

Supplementary Figure S16: Normalized DIC assimilation rate of live *Thyasira tokunagai*. Data are averaged from six treatments and incubated at *in situ* temperature, from a total of eight stations.

Fig. S17.

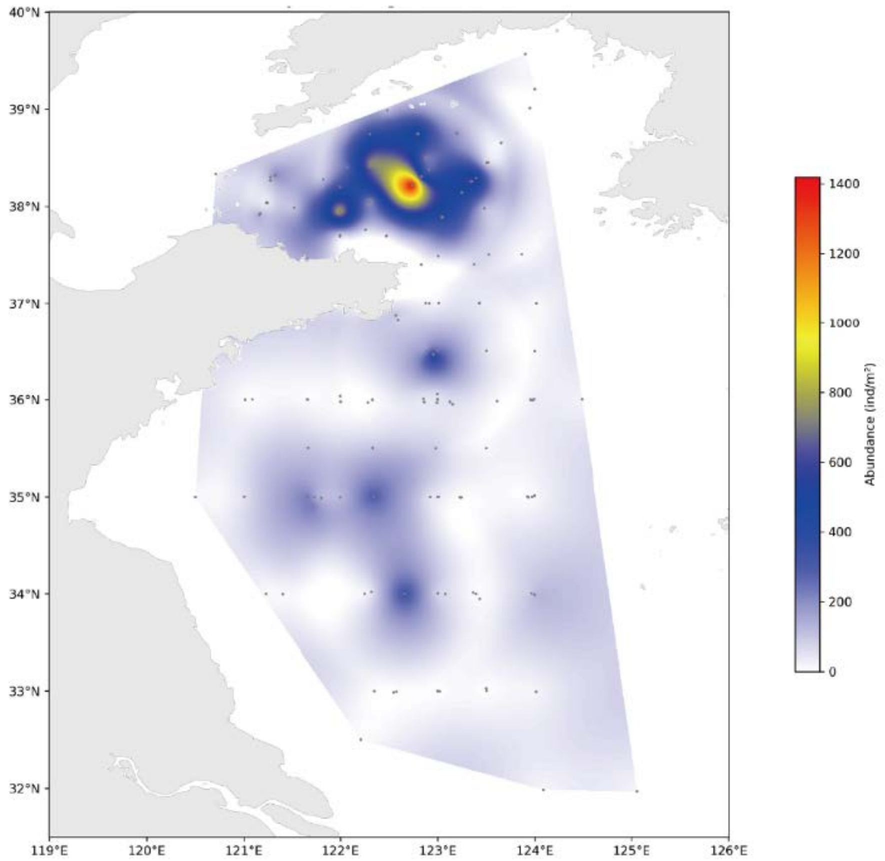

Supplementary Figure S17: *Thyasira tokunagai* predicted abundance. Based on data from 162 stations across nine cruises, we predicted clam abundance using the kriging interpolation method within sampling area for carbon fixation flux estimation.

Legends of Supplementary Tables:

Supplementary Table S1: *Thyasira tokunagai* abundance and general environmental parameters at each sampling station.

Supplementary Table S2: Details of *Thyasira tokunagai* specimens used in this study, including the type of analysis performed.

Supplementary Table S3: Table S3: Stable isotope information used in this study from the Yellow Sea.

Supplementary Table S4: Statistics for *Thyasira tokunagai* symbiont genome assembly and binning using combined long-read and short-read sequencing data.

Supplementary Table S5: Statistics for average nucleotide identity (ANI) matrix of thirty symbiont genomes.

Supplementary Table S6: Difference of functional coding genes between two symbiont phylotypes.

Supplementary Table S7: All concatenated genes and nucleotide positions used for building the symbiont phylogenetic tree.

Supplementary Table S8: Metabolism capacity of symbiont to biosynthesize amnio acids, vitamins, and cofactors.

Supplementary Table S9: Measurement records of DIC assimilation rate using radioactive carbon tracing.

Supplementary Table S10: Annotation of functional coding genes within the pangenome inferred from thirty symbiont genomes.

Supplementary Table S11: Metadata associated with *cox1* gene sequences used to construct the phylogenetic tree of *Thyasira tokunagai*.

Legend of Supplementary Commands:

Bioinformatic commands used in this study

REFERENCES

1. J. Bascompte, Mutualism and biodiversity. *Curr. Biol.* **29**, R467–R470 (2019).

2. R. M. Fisher, L. M. Henry, C. K. Cornwallis, E. T. Kiers, S. A. West, The evolution of host-symbiont dependence. *Nat. Commun.* **8**, 15973 (2017).

3. G. M. Bennett, N. A. Moran, Heritable symbiosis: The advantages and perils of an evolutionary rabbit hole. *Proc. Natl. Acad. Sci. U.S.A.* **112**, 10169–10176 (2015).

4. C. K. Cornwallis, A. van 't Padje, J. Ellers, M. Klein, R. Jackson, E. T. Kiers, S. A. West, L. M. Henry, Symbioses shape feeding niches and diversification across insects. *Nat. Ecol. Evol.* **7**, 1022–1044 (2023).

5. N. Dubilier, C. Bergin, C. Lott, Symbiotic diversity in marine animals: The art of harnessing chemosynthesis. *Nat. Rev. Microbiol.* **6**, 725–740 (2008).

6. T. Hinzke, M. Kleiner, C. Breusing, H. Felbeck, R. Häsler, M. Sievert Stefan, R. Schlüter, P. Rosenstiel, B. H. Reusch Thorsten, T. Schweder, S. Markert, Host-microbe interactions in the chemosynthetic *Riftia pachyptila* symbiosis. *MBio* **10**, e02243-19 (2019).

7. Y. Yang, J. Sun, Y. Sun, Y. H. Kwan, W. C. Wong, Y. Zhang, T. Xu, D. Feng, Y. Zhang, J.-W. Qiu, P.-Y. Qian, Genomic, transcriptomic, and proteomic insights into the symbiosis of deep-sea tubeworm holobionts. *ISME J.* **14**, 135–150 (2020).

8. Y. Sun, J. Sun, Y. Yang, Y. Lan, J. C.-H. Ip, W. C. Wong, Y. H. Kwan, Y. Zhang, Z. Han, J.-W. Qiu, P.-Y. Qian, Genomic signatures supporting the symbiosis and formation of chitinous tube in the deep-sea tubeworm *Paraescarpia echinospica*. *Mol. Biol. Evol.* **38**, 4116–4134 (2021).

9. Y. Lan, J. Sun, C. Chen, H. Wang, Y. Xiao, M. Perez, Y. Yang, Y. H. Kwan, Y. Sun, Y. Zhou, X. Han, J. Miyazaki, T.-o. Watsuji, D. Bissessur, J.-W. Qiu, K. Takai, P.-Y. Qian, Endosymbiont population genomics sheds light on transmission mode, partner specificity, and stability of the scaly-foot snail holobiont. *ISME J.* **16**, 2132–2143 (2022).

10. C. Breusing, J. Mitchell, J. Delaney, S. P. Sylva, J. S. Seewald, P. R. Girguis, R. A. Beinart, Physiological dynamics of chemosynthetic symbionts in hydrothermal vent snails. *ISME J.* **14**, 2568–2579 (2020).

11. Y. Lan, J. Sun, C. Chen, Y. Sun, Y. Zhou, Y. Yang, W. Zhang, R. Li, K. Zhou, W. C. Wong, Y. H. Kwan, A. Cheng, S. Bougouffa, C. L. Van Dover, J.-W. Qiu, P.-Y. Qian, Hologenome analysis reveals dual symbiosis in the deep-sea hydrothermal vent snail *Gigantopelta aegis*. *Nat. Commun.* **12**, 1165 (2021).

12. M. Franke, B. Geier, J. U. Hammel, N. Dubilier, N. Leisch, Coming together—Symbiont acquisition and early development in deep-sea bathymodioline mussels. *Proc. R. Soc. B* **288**, 20211044 (2021).

13. J. Sun, Y. Zhang, T. Xu, Y. Zhang, H. Mu, Y. Zhang, Y. Lan, C. J. Fields, J. H. L. Hui, W. Zhang, R. Li, W. Nong, F. K. M. Cheung, J.-W. Qiu, P.-Y. Qian, Adaptation to deep-sea chemosynthetic environments as revealed by mussel genomes. *Nat. Ecol. Evol.* **1**, 0121 (2017).

14. J. C.-H. Ip, T. Xu, J. Sun, R. Li, C. Chen, Y. Lan, Z. Han, H. Zhang, J. Wei, H. Wang, J. Tao, Z. Cai, P.-Y. Qian, J.-W. Qiu, Host-endosymbiont genome integration in a deep-sea chemosymbiotic clam. *Mol. Biol. Evol.* **38**, 502–518 (2020).

15. Y. Li, X. He, Y. Lin, Y.-X. Li, G. M. Kamenev, J. Li, J.-W. Qiu, J. Sun, Reduced chemosymbiont genome in the methane seep thyasirid and the cooperated metabolisms in the holobiont under anaerobic sediment. *Mol. Ecol. Resour.* **23**, 1853–1867 (2023).

16. E. M. Sogin, N. Leisch, N. Dubilier, Chemosynthetic symbioses. *Curr. Biol.* **30**, R1137–R1142 (2020).

17. I. Killeen, P. G. Oliver, The taxonomic and conservation status of *Thyasira gouldi* (Philippi, 1844), the northern hatchet shell in British waters. *J. Conchol.* **37**, 391–402 (2002).

18. Y.-R. Kim, S. Lee, J. Kim, C.-J. Kim, K.-Y. Choi, C.-S. Chung, *Thyasira tokunagai* as an ecological indicator for the quality of sediment and benthic communities in the East Sea-Byeong, Korea. *Mar. Pollut. Bull.* **135**, 873–879 (2018).

19. P. G. Oliver, A. M. Holmes, New species of Thyasiridae (Bivalvia) from chemosynthetic communities in the Atlantic Ocean. *J. Conchol.* **39**, 175–184 (2006).

20. E. K. Åström, P. G. Oliver, M. L. Carroll, A new genus and two new species of Thyasiridae associated with methane seeps off Svalbard, Arctic Ocean. *Mar. Biol. Res.* **13**, 402–416 (2017).

21. K. Fujikura, S. Kojima, K. Tamaki, Y. Maki, J. Hunt, T. Okutani, The deepest chemosynthesis-based community yet discovered from the hadal zone, 7326 m deep, in the Japan Trench. *Mar. Ecol. Prog. Ser.* **190**, 17–26 (1999).

22. X. Peng, M. Du, A. Gebruk, S. Liu, Z. Gao, R. N. Glud, P. Zhou, R. Wang, A. A. Rowden, G. M. Kamenev, A. S. Maiorova, D. Papineau, S. Chen, J. Gao, H. Liu, Y. He, I. L. Alalykina, I. Y. Dolmatov, H. Zhang, X. Li, M. V. Malyutina, S. Dasgupta, A. A. Saulenko, V. A. Shilov, S. Liu, T. Xie, Y. Qu, X. Song, H. Zhang, H. Liu, W. Zhang, X. Huang, H. Xu, W. Xu, V. V. Mordukhovich, A. V. Adrianov, Flourishing chemosynthetic life at the greatest depths of hadal trenches. *Nature* **645**, 679–685 (2025).

23. R. T. Batstone, J. R. Laurich, F. Salvo, S. C. Dufour, Divergent chemosymbiosis-related characters in *Thyasira* cf. *gouldi* (Bivalvia: Thyasiridae). *PLOS ONE* **9**, e92856 (2014).

24. R. Dove, B. McCuaig, M. Giolland, V. Kokarev, S. C. Dufour, Taxonomic and biological remarks on *Thyasira gouldii* (Philippi, 1845) in Eastern Canada and reinstatement of the species *Thyasira plana* (Verrill and Bush, 1898). *Am. Malacol. Bull.* **42**, 1–17 (2025).

25. S. C. Dufour, H. Felbeck, Sulphide mining by the superextensile foot of symbiotic thyasirid bivalves. *Nature* **426**, 65–67 (2003).

26. S. C. Dufour, J. R. Laurich, R. T. Batstone, B. McCuaig, A. Elliott, K. M. Poduska, Magnetosome-containing bacteria living as symbionts of bivalves. *ISME J.* **8**, 2453–2462 (2014).

27. S. C. Dufour, Gill anatomy and the evolution of symbiosis in the bivalve family Thyasiridae. *Biol. Bull.* **208**, 200–212 (2005).

28. B. McCuaig, F. Liboiron, S. C. Dufour, The bivalve *Thyasira* cf. *gouldi* hosts chemoautotrophic symbiont populations with strain level diversity. *PeerJ* **5**, e3597 (2017).

29. Y. Fujiwara, K. Fujikura, S. Kojima, C. Kato, N. Masui, Dual symbiosis in the cold-seep thyasirid clam *Maorithyas hadalis* from the hadal zone in the Japan Trench, western Pacific. *Mar. Ecol. Prog. Ser.* **214**, 151–159 (2001).

30. Y. Xu, J. Sui, L. Ma, X. Li, H. Wang, B. Zhang, Temporal variation of macrobenthic community zonation over nearly 60 years and the effects of latitude and depth in the southern Yellow Sea and East China Sea. *Sci. Total Environ.* **739**, 139760 (2020).

31. J. R. Laurich, R. Dove, C. Paillard, S. C. Dufour, Life and death in facultative chemosymbioses: Control of bacterial population dynamics in the Thyasiridae. *Symbiosis* **75**, 123–133 (2018).

32. X. S. Liu, D. P. Ni, X. Zhong, Z. N. Zhang, Structure of benthic foot web and trophic relationship of macrofauna in the Yellow Sea. *Period. Ocean Univ. China* **50**, 20–33 (2020).

33. C. Jain, L. M. Rodriguez-R, A. M. Phillippy, K. T. Konstantinidis, S. Aluru, High throughput ANI analysis of 90K prokaryotic genomes reveals clear species boundaries. *Nat. Commun.* **9**, 5114 (2018).

34. B. McCuaig, L. Peña-Castillo, S. C. Dufour, Metagenomic analysis suggests broad metabolic potential in extracellular symbionts of the bivalve *Thyasira* cf. *gouldi*. *Anim. Microbiome* **2**, 7 (2020).

35. M. Rubin-Blum, N. Dubilier, M. Kleiner, Genetic evidence for two carbon fixation pathways (the Calvin-Benson-Bassham Cycle and the Reverse Tricarboxylic Acid Cycle) in symbiotic and free-living bacteria. *mSphere* **4**, e00394-18 (2019).

36. H. Nomaki, C. Chen, N. Ogawa, Y. Miyairi, N. Ohkouchi, A. Makabe, S. Kawagucci, Y. Yokoyama, M. Shimanaga, Elucidating carbon sources of hydrothermal vent animals using natural ^{14}C abundances and habitat water temperature. *Limnol. Oceanogr.* **69**, 1270–1284 (2024).

37. H. Zanzerl, F. Salvo, S. W. Jones, S. C. Dufour, Feeding strategies in symbiotic and asymbiotic thyasirid bivalves. *J. Sea Res.* **145**, 16–23 (2019).

38. J. J. Robinson, C. M. Cavanaugh, Expression of form I and form II Rubisco in chemoautotrophic symbioses: Implications for the interpretation of stable carbon isotope values. *Limnol. Oceanogr.* **40**, 1496–1502 (1995).

39. U. Cardini, M. Bartoli, S. Lücker, M. Mooshammer, J. Polzin, R. W. Lee, V. Mičić, T. Hofmann, M. Weber, J. M. Petersen, Chemosymbiotic bivalves contribute to the nitrogen budget of seagrass ecosystems. *ISME J.* **13**, 3131–3134 (2019).

40. E. M. Sogin, M. Kleiner, C. Borowski, H. R. Gruber-Vodicka, N. Dubilier, Life in the dark: Phylogenetic and physiological diversity of chemosynthetic symbioses. *Annu. Rev. Microbiol.* **75**, 695–718 (2021).

41. J. Zhang, R. Liu, S. Xi, R. Cai, X. Zhang, C. Sun, A novel bacterial thiosulfate oxidation pathway provides a new clue about the formation of zero-valent sulfur in deep sea. *ISME J.* **14**, 2261–2274 (2020).

42. L. H. Gregersen, D. A. Bryant, N.-U. Frigaard, Mechanisms and evolution of oxidative sulfur metabolism in green sulfur bacteria. *Front. Microbiol.* **2**, 116 (2011).

43. S. C. Dufour, H. Felbeck, Symbiont abundance in thyasirids (Bivalvia) is related to particulate food and sulphide availability. *Mar. Ecol. Prog. Ser.* **320**, 185–194 (2006).

44. K. Anantharaman, M. B. Duhaime, J. A. Breier, K. A. Wendt, B. M. Toner, G. J. Dick, Sulfur oxidation genes in diverse deep-sea viruses. *Science* **344**, 757–760 (2014).

45. J. M. Klatt, L. Polerecky, Assessment of the stoichiometry and efficiency of CO_2 fixation coupled to reduced sulfur oxidation. *Front. Microbiol.* **6**, 484 (2015).

46. G. F. Paredes, T. Viehboeck, R. Lee, M. Palatinszky, M. A. Mausz, S. Reipert, A. Schintlmeister, A. Maier, J.-M. Volland, C. Hirschfeld, M. Wagner, D. Berry, S. Markert, S. Bulgheresi, L. König, Anaerobic sulfur oxidation underlies adaptation of a chemosynthetic symbiont to oxic-anoxic interfaces. *mSystems* **6**, e0118620 (2021).

47. T. J. Hackmann, Setting new boundaries of 16S rRNA gene identity for prokaryotic taxonomy. *Int. J. Syst. Evol. Microbiol.* **75**, 006747 (2025).

48. R. Ansorge, S. Romano, L. Sayavedra, M. Á. G. Porras, A. Kupczok, H. E. Tegetmeyer, N. Dubilier, J. Petersen, Functional diversity enables multiple symbiont strains to coexist in deep-sea mussels. *Nat. Microbiol.* **4**, 2487–2497 (2019).

49. S. Duperron, A. Quiles, K. M. Szafranski, N. Leger, B. Shillito, Estimating symbiont abundances and gill surface areas in specimens of the hydrothermal vent mussel *Bathymodiolus puteoserpentis* maintained in pressure vessels. *Front. Mar. Sci.* **3**, 16 (2016).

50. J. M. Petersen, F. U. Zielinski, T. Pape, R. Seifert, C. Moraru, R. Amann, S. Hourdez, P. R. Girguis, S. D. Wankel, V. Barbe, E. Pelletier, D. Fink, C. Borowski, W. Bach, N. Dubilier, Hydrogen is an energy source for hydrothermal vent symbioses. *Nature* **476**, 176–180 (2011).

51. M. Scott Kathleen, M. Cavanaugh Colleen, CO_2 uptake and fixation by endosymbiotic chemoautotrophs from the Bivalve *Solemya velum*. *Appl. Environ. Microbiol.* **73**, 1174–1179 (2007).

52. B. Liu, Y. Zheng, X. Wang, L. Qi, J. Zhou, Z. An, L. Wu, F. Chen, Z. Lin, G. Yin, H. Dong, X. Li, X. Liang, P. Han, M. Liu, L. Hou, Active dark carbon fixation evidenced by ^{14}C isotope assimilation and metagenomic data across the estuarine-coastal continuum. *Sci. Total Environ.* **914**, 169833 (2024).

53. T. Xu, Y. Sun, Z. Wang, A. Sen, P.-Y. Qian, J.-W. Qiu, The morphology, mitogenome, phylogenetic position, and symbiotic bacteria of a new species of *Sclerolinum* (Annelida: Siboglinidae) in the South China Sea. *Front. Mar. Sci.* **8**, 793645 (2022).

54. W. Bernhard, A new staining procedure for electron microscopical cytology. *J. Ultrastruct. Res.* **27**, 250–265 (1969).

55. H. Daims, A. Brühl, R. Amann, K.-H. Schleifer, M. Wagner, The domain-specific probe EUB338 is insufficient for the detection of all bacteria: Development and evaluation of a more comprehensive probe set. *Syst. Appl. Microbiol.* **22**, 434–444 (1999).

56. J. A. Frank, C. I. Reich, S. Sharma, J. S. Weisbaum, B. A. Wilson, G. J. Olsen, Critical evaluation of two primers commonly used for amplification of bacterial 16S rRNA genes. *Appl. Environ. Microbiol.* **74**, 2461–2470 (2008).

57. D. P. Herlemann, M. Labrenz, K. Jürgens, S. Bertilsson, J. J. Waniek, A. F. Andersson, Transitions in bacterial communities along the 2000 km salinity gradient of the Baltic Sea. *ISME J.* **5**, 1571–1579 (2011).

58. J. G. Caporaso, J. Kuczynski, J. Stombaugh, K. Bittinger, F. D. Bushman, E. K. Costello, N. Fierer, A. G. Peña, J. K. Goodrich, J. I. Gordon, G. A. Huttley, S. T. Kelley, D. Knights, J. E. Koenig, R. E. Ley, C. A. Lozupone, D. McDonald, B. D. Muegge, M. Pirrung, J. Reeder, J. R. Sevinsky, P. J. Turnbaugh, W. A. Walters, J. Widmann, T. Yatsunenko, J. Zaneveld, R. Knight, QIIME allows analysis of high-throughput community sequencing data. *Nat. Methods* **7**, 335–336 (2010).

59. R. R. Wick, L. M. Judd, K. E. Holt, Performance of neural network basecalling tools for Oxford Nanopore sequencing. *Genome Biol.* **20**, 129 (2019).

60. A. Chen, S. Liao, M. Cheng, K. Ma, L. Wu, Y. Lai, X. Qiu, J. Yang, J. Xu, S. Hao, Spatiotemporal transcriptomic atlas of mouse organogenesis using DNA nanoball-patterned arrays. *Cell* **185**, 1777–1792 (2022).

61. A. M. Bolger, M. Lohse, B. Usadel, Trimmomatic: A flexible trimmer for Illumina sequence data. *Bioinformatics* **30**, 2114–2120 (2014).

62. D. Li, C.-M. Liu, R. Luo, K. Sadakane, T.-W. Lam, MEGAHIT: An ultra-fast single-node solution for large and complex metagenomics assembly via succinct *de Bruijn* graph. *Bioinformatics* **31**, 1674–1676 (2015).

63. Y.-W. Wu, B. A. Simmons, S. W. Singer, MaxBin 2.0: An automated binning algorithm to recover genomes from multiple metagenomic datasets. *Bioinformatics* **32**, 605–607 (2015).

64. D. Laetsch, M. Blaxter, BlobTools: Interrogation of genome assemblies. *F1000Res* **6**, 1287 (2017).

65. J. Hu, Z. Wang, Z. Sun, B. Hu, A. O. Ayoola, F. Liang, J. Li, J. R. Sandoval, D. N. Cooper, K. Ye, J. Ruan, C.-L. Xiao, D. Wang, D.-D. Wu, S. Wang, NextDenovo: An efficient error correction and accurate assembly tool for noisy long reads. *Genome Biol.* **25**, 107 (2024).

66. J. Hu, J. Fan, Z. Sun, S. Liu, NextPolish: A fast and efficient genome polishing tool for long-read assembly. *Bioinformatics* **36**, 2253–2255 (2019).

67. A. Chklovski, D. H. Parks, B. J. Woodcroft, G. W. Tyson, CheckM2: A rapid, scalable and accurate tool for assessing microbial genome quality using machine learning. *Nat. Methods* **20**, 1203–1212 (2023).

68. P.-A. Chaumeil, A. J. Mussig, P. Hugenholtz, D. H. Parks, GTDB-Tk v2: Memory friendly classification with the genome taxonomy database. *Bioinformatics* **38**, 5315–5316 (2022).

69. T. Seemann, Prokka: Rapid prokaryotic genome annotation. *Bioinformatics* **30**, 2068–2069 (2014).

70. B. Buchfink, C. Xie, D. H. Huson, Fast and sensitive protein alignment using DIAMOND. *Nat. Methods* **12**, 59–60 (2015).

71. A. Conesa, S. Götz, J. M. García-Gómez, J. Terol, M. Talón, M. Robles, Blast2GO: A universal tool for annotation, visualization and analysis in functional genomics research. *Bioinformatics* **21**, 3674–3676 (2005).

72. M. Y. Galperin, Y. I. Wolf, K. S. Makarova, R. Vera Alvarez, D. Landsman, E. V. Koonin, COG database update: Focus on microbial diversity, model organisms, and widespread pathogens. *Nucleic Acids Res.* **49**, D274–D281 (2020).

73. M. Kanehisa, Y. Sato, K. Morishima, BlastKOALA and GhostKOALA: KEGG tools for functional characterization of genome and metagenome sequences. *J. Mol. Biol.* **428**, 726–731 (2016).

74. E. D. Graham, J. F. Heidelberg, B. J. Tully, Potential for primary productivity in a globally-distributed bacterial phototroph. *ISME J.* **12**, 1861–1866 (2018).

75. Z. Gu, Complex heatmap visualization. *iMeta* **1**, e43 (2022).

76. F. Beghini, L. J. McIver, A. Blanco-Míguez, L. Dubois, F. Asnicar, S. Maharjan, A. Mailyan, P. Manghi, M. Scholz, A. M. Thomas, M. Valles-Colomer, G. Weingart, Y. Zhang, M. Zolfo, C. Huttenhower, E. A. Franzosa, N. Segata, Integrating taxonomic, functional, and strain-level profiling of diverse microbial communities with bioBakery 3. *eLife* **10**, e65088 (2021).

77. H. Hu, Y. Tan, C. Li, J. Chen, Y. Kou, Z. Z. Xu, Y. Y. Liu, Y. Tan, L. Dai, StrainPanDA: Linked reconstruction of strain composition and gene content profiles via pangenome-based decomposition of metagenomic data. *iMeta* **1**, e41 (2022).

78. D. M. Emms, Y. Liu, L. Belcher, J. Holmes, S. Kelly, OrthoFinder: Scalable phylogenetic orthology inference for comparative genomics. bioRxiv 664860 [Preprint] (2025); https://doi.org/10.1101/2025.07.15.664860.

79. C. P. Cantalapiedra, A. Hernández-Plaza, I. Letunic, P. Bork, J. Huerta-Cepas, eggNOG-mapper v2: Functional annotation, orthology assignments, and domain prediction at the metagenomic scale. *Mol. Biol. Evol.* **38**, 5825–5829 (2021).

80. Y. Li, X. Liu, C. Chen, J.-W. Qiu, K. Kocot, J. Sun, VEHoP: A versatile, Easy-to-use, and Homology-based Phylogenomic pipeline accommodating diverse sequences. bioRxiv 604968 [Preprint] (2024); https://doi.org/10.1101/2024.07.24.604968.

81. J. Xie, Y. Chen, G. Cai, R. Cai, Z. Hu, H. Wang, Tree Visualization By One Table (tvBOT): A web application for visualizing, modifying and annotating phylogenetic trees. *Nucleic Acids Res.* **51**, W587–W592 (2023).

82. B. Langmead, S. L. Salzberg, Fast gapped-read alignment with Bowtie 2. *Nat. Methods* **9**, 357–359 (2012).

83. M. G. Grabherr, B. J. Haas, M. Yassour, J. Z. Levin, D. A. Thompson, I. Amit, X. Adiconis, L. Fan, R. Raychowdhury, Q. Zeng, Full-length transcriptome assembly from RNA-Seq data without a reference genome. *Nat. Biotechnol.* **29**, 644–652 (2011).

84. B. Haas, A. Papanicolaou, TransDecoder (find coding regions within transcripts). Google Scholar (2016).

85. R. Patro, G. Duggal, M. I. Love, R. A. Irizarry, C. Kingsford, Salmon provides fast and bias-aware quantification of transcript expression. *Nat. Methods* **14**, 417–419 (2017).

86. G.-C. Zhuang, A. Montgomery, S. B. Joye, Heterotrophic metabolism of C1 and C2 low molecular weight compounds in northern Gulf of Mexico sediments: Controlling factors and implications for organic carbon degradation. *Geochim. Cosmochim. Acta* **247**, 243–260 (2019).

87. S.-H. Mao, Z. Zhou, S.-B. Yan, G.-B. Xu, X.-J. Li, H.-H. Zhang, G.-P. Yang, G.-C. Zhuang, Microbial metabolism and environmental controls of acetate cycling in the northwest Pacific Ocean. *Geophys. Res. Lett.* **51**, e2024GL109692 (2024).

88. G. C. Zhuang, T. D. Peña-Montenegro, A. Montgomery, J. P. Montoya, S. B. Joye, Significance of acetate as a microbial carbon and energy source in the water column of Gulf of Mexico: Implications for marine carbon cycling. *Global Biogeochem. Cycles* **33**, 223–235 (2019).

89. N. Dierckxsens, P. Mardulyn, G. Smits, NOVOPlasty: De novo assembly of organelle genomes from whole genome data. *Nucleic Acids Res.* **45**, e18 (2016).

90. M. Bernt, A. Donath, F. Jühling, F. Externbrink, C. Florentz, G. Fritzsch, J. Pütz, M. Middendorf, P. F. Stadler, MITOS: Improved de novo metazoan mitochondrial genome annotation. *Mol. Phylogenet. Evol.* **69**, 313–319 (2013).

91. R. C. Edgar, MUSCLE: Multiple sequence alignment with high accuracy and high throughput. *Nucleic Acids Res.* **32**, 1792–1797 (2004).

92. J. Castresana, Selection of conserved blocks from multiple alignments for their use in phylogenetic analysis. *Mol. Biol. Evol.* **17**, 540–552 (2000).

93. S. Kumar, G. Stecher, M. Li, C. Knyaz, K. Tamura, MEGA X: Molecular evolutionary genetics analysis across computing platforms. *Mol. Biol. Evol.* **35**, 1547–1549 (2018).

94. J. Rozas, A. Ferrer-Mata, J. C. Sánchez-DelBarrio, S. Guirao-Rico, P. Librado, S. E. Ramos-Onsins, A. Sánchez-Gracia, DnaSP 6: DNA sequence polymorphism analysis of large data sets. *Mol. Biol. Evol.* **34**, 3299–3302 (2017).

95. J. W. Leigh, D. Bryant, POPART: Full-feature software for haplotype network construction. *Methods Ecol. Evol.* **6**, 1110–1116 (2015).

96. K. Katoh, K. Misawa, K. i. Kuma, T. Miyata, MAFFT: A novel method for rapid multiple sequence alignment based on fast fourier transform. *Nucleic Acids Res.* **30**, 3059–3066 (2002).

97. D. Falush, M. Stephens, J. K. Pritchard, Inference of population structure using multilocus genotype data: Linked loci and correlated allele frequencies. *Genetics* **164**, 1567–1587 (2003).

98. D. A. Earl, B. M. vonHoldt, STRUCTURE HARVESTER: A website and program for visualizing STRUCTURE output and implementing the Evanno method. *Conserv. Genet. Resour.* **4**, 359–361 (2012).

99. S. Müller, L. Schüler, A. Zech, F. Heße, GSTools v1.3: A toolbox for geostatistical modelling in Python. *Geosci. Model Dev.* **15**, 3161–3182 (2022).

100. C. Prentice, M. Hessing-Lewis, R. Sanders-Smith, A. K. Salomon, Reduced water motion enhances organic carbon stocks in temperate eelgrass meadows. *Limnol. Oceanogr.* **64**, 2389–2404 (2019).

101. F. Wang, J. Liu, G. Qin, J. Zhang, J. Zhou, J. Wu, L. Zhang, P. Thapa, C. J. Sanders, I. R. Santos, X. Li, G. Lin, Q. Weng, J. Tang, N. Jiao, H. Ren, Coastal blue carbon in China as a nature-based solution toward carbon neutrality. *Int. J. Hydrog Energ.* **4**, 100481 (2023).
